# Harms in Systematic Reviews Paper 2: Methods used to assess harms are neglected in systematic reviews of gabapentin

**DOI:** 10.1016/j.jclinepi.2021.10.024

**Published:** 2021-11-03

**Authors:** Riaz Qureshi, Evan Mayo-Wilson, Thanitsara Rittiphairoj, Mara McAdams-DeMarco, Eliseo Guallar, Tianjing Li

**Affiliations:** a Department of Epidemiology, Johns Hopkins Bloomberg School of Public Health, Baltimore, MD, USA; b Department of Epidemiology and Biostatistics, Indiana University School of Public Health, Bloomington, ID, USA; c Cochrane Eyes and Vision United States, University of Colorado Anschutz Medical Campus, Aurora, CO, USA; d Department of Surgery, Department of Epidemiology, Johns Hopkins School of Medicine and Bloomberg School of Public Health, Baltimore, MD, USA; e Department of Epidemiology, Johns Hopkins Bloomberg School of Public Health, Baltimore, MD, USA; f Department of Ophthalmology, University of Colorado Anschutz Medical Campus, Aurora, CO, USA

**Keywords:** Harms, Systematic Reviews, Meta-analysis, Synthesis, Clinical Trials

## Abstract

**Objective:**

We compared methods used with current recommendations for synthesizing harms in systematic reviews and meta-analyses (SRMAs) of gabapentin.

**Study Design & Setting:**

We followed recommended systematic review practices. We selected reliable SRMAs of gabapentin (i.e., met a pre-defined list of methodological criteria) that assessed at least one harm. We extracted and compared methods in four areas: pre-specification, searching, analysis, and reporting. Whereas our focus in this paper is on the methods used, Part 2 examines the results for harms across reviews.

**Results:**

We screened 4320 records and identified 157 SRMAs of gabapentin, 70 of which were reliable. Most reliable reviews (51/70; 73%) reported following a general guideline for SRMA conduct or reporting, but none reported following recommendations specifically for synthesizing harms. Across all domains assessed, review methods were designed to address questions of benefit and rarely included the additional methods that are recommended for evaluating harms.

**Conclusion:**

Approaches to assessing harms in SRMAs we examined are tokenistic and unlikely to produce valid summaries of harms to guide decisions. A paradigm shift is needed. At a minimal, reviewers should describe any limitations to their assessment of harms and provide clearer descriptions of methods for synthesizing harms.

## 1. Background

Systematic reviews of randomized controlled trials are often considered the pinnacle of the evidence pyramid for answering research questions related to effectiveness.[1] Guidelines recommend that potential harms (Box 1) be assessed alongside potential benefits to avoid one-sided summaries of evidence.[2] A given systematic review might take one of three approaches to assessing harms: pre-specifying all harms of interest, not pre-specifying any harms, or a hybrid approach (Box 1).[3] The choice of approach, might depend of the intervention and setting, which can dictate whether an outcome is treated as a potential harm or benefit. For example, weight gain is considered a harm in trials of antipsychotics but might be a benefit in trials of interventions for eating disorders. These approaches have complementary strengths and weaknesses.[3]

Meta-research has shown that primary studies and systematic reviews use poor methods to assess harms and report them poorly.[3,4,13–21,5–12] Guidance on how to synthesize harms is summarized in Box 2,[2,7–9,22] and paper 1 of this series provides an introduction to these issues.[23]

Our objectives in this study were to assess whether reviews: used methods described in these guidelines, used appropriate sources of evidence, and applied appropriate methods to synthesize harms. We compared results for harms in a second paper.[24]

We selected gabapentin as a case example because it was likely that there would be multiple systematic reviews to compare Additionally, gabapentin is used widely for a range of conditions, of which most prescriptions are off-label.[25]

## 2. Methods

We prespecified eligibility criteria, the search strategy, items for extraction, and the comparison of methods with recommendations. As part of a PhD dissertation proposal, the protocol was reviewed by a committee and is attached as APPENDIX A. We screened search results independently in duplicate. We report relevant items following the PRISMA 2020.[26]

### 2.1. Selection of reviews

To be included in our study, we required that reviews: (i) be systematic reviews or meta-analyses; (ii) examine gabapentin for one of its commonly prescribed conditions(on- or off-label), including: alcohol dependence, epilepsy, pain (postherpetic neuralgia, neuropathic pain, post-operative pain, fibromyalgia, migraine), psychiatric disorders (bipolar disorder, attention deficit disorder, obsessive compulsive disorder, and post-traumatic stress disorder), restless leg syndrome, and vasomotor symptoms, and; (iii) have any results for harms (which could have included a general statement that no harms were reported in the included studies); and (iv) be reliable in methods (i.e., met a pre-defined list of methodological criteria). We defined systematic reviews as articles that either (i) self-identified as a systematic review or metanalysis, or (ii) followed a structured methodology to synthesize research, as per the Institute of Medicine’s definition of a systematic review.[7]

We focused on reliable reviews, which would be the “best case scenario” for agreement in methods for assessing and reporting harms. We excluded reviews published only as abstracts or for which a full text was unavailable because it is not possible to assess the reliability or methods of a review based solely on an abstract.

### 2.2. Search methods for identification of reviews

We searched PubMed, EMBASE, Epistemonikos, and the Cochrane Database of Systematic Reviews from 1990 to September 17, 2020 without any language restrictions (gabapentin was first approved for use by the United States Food and Drug Administration in 1993). We developed a search strategy with informationists from Johns Hopkins Welch Medical library (APPENDIX B).

We used EndNote then Covidence for de-duplication and screening. Two authors (RQ and TR) screened titles and abstracts, and full texts, independently and resolved disagreements through discussion. We piloted the screening with 100 and 50 records at the title/abstract and full-text levels, respectively.

### 2.3. Data extraction

We extracted all details about the methods used in systematic reviews to assess harms, grouped as follows: approach/planning, searching, analysis, and reporting. Specific items extracted for each of these domains can be found in APPENDIX A. We extracted these data from only studies that we considered to be reliable based on methodologic criteria developed by Cochrane Eyes and Vision United States Satellite (APPENDIX C).[27–32]

Both reliability assessment and data extraction were done by two reviewers using single extraction with verification, which isas accurate as independent data extraction but less resource intensive.[33] We used the Systematic Review Data Repository to extract the data from all included reviews.[34] We did not formally pilot test the data extraction form; the structure and many items were taken from previously developed forms and the reliability assessment has been used many times.[27–32]

### 2.4. Analysis and synthesis

We qualitatively describe and compare the methods used for harms across all studies that met our inclusion criteria. We performed two *post hoc* subgroup analyses based on (i) whether reviews pre-specified any harms and (ii) whether the primary purpose of the review was to assess questions related to harms or benefits. Reviews with pre-specified harms might have included a search for studies of those harms. By contrast, reviews focused on potential benefits might not include a search for harms at all. Thus, we explored differences in the searches and types of evidence used to address these different types of research questions.

We used Microsoft Excel and Stata 15 to tabulate all results.

## 3. Results

### 3.1. Selection of reviews

**Fig. 1.**
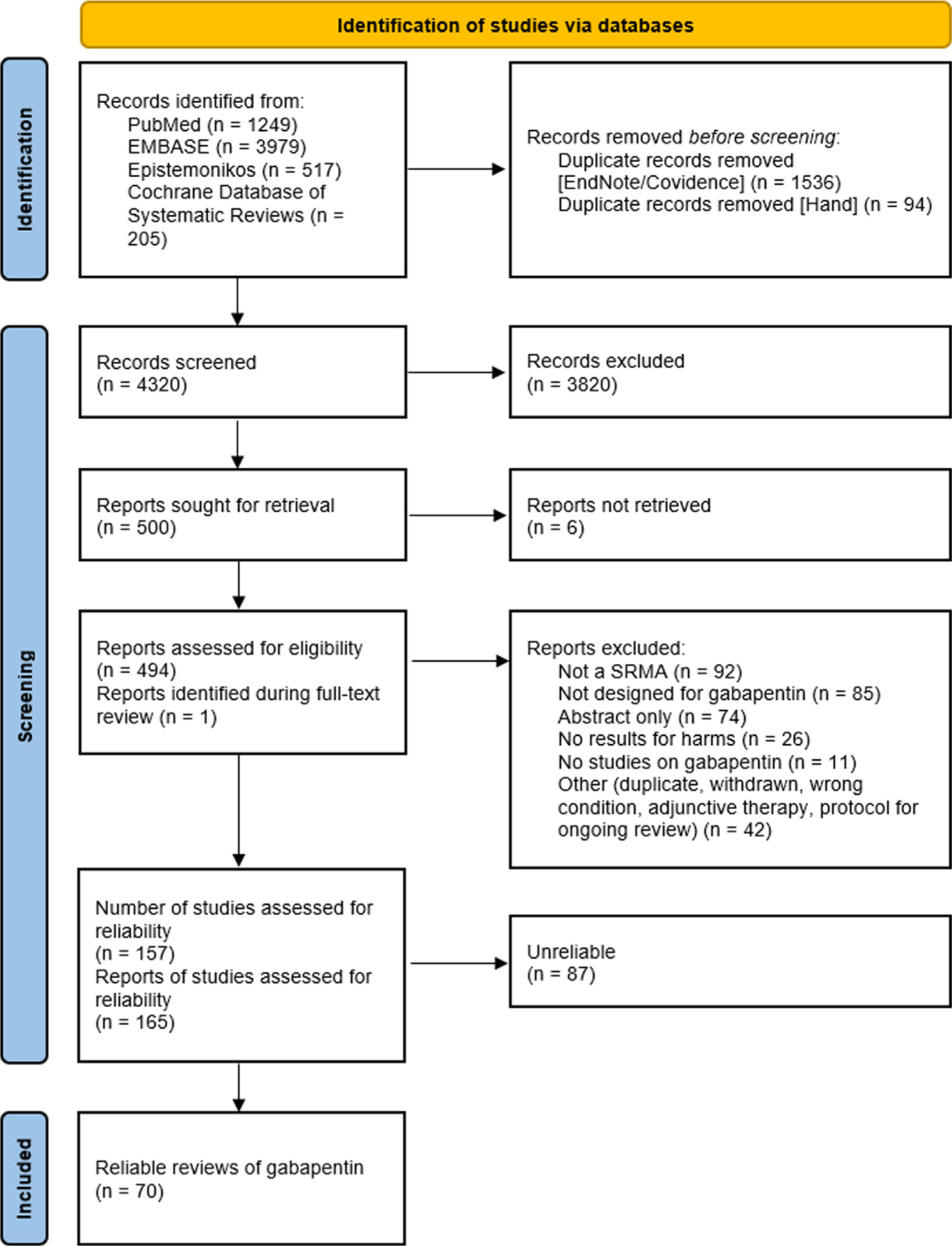
Study selection flow diagram.

Fig. 1 depicts the study selection flow diagram. We identified 4320 unique records, reviewed 500 full-text reports, and ultimately included 165 records for 157 reviews (Fig. 1). Of the 157 reviews, 70 were considered reliable 87 unreliable reviews were excluded from further analysis.

### 3.2. Characteristics of included reviews

Table 1A presents the general characteristics of included reviews. Most reviews (61/70; 87%) were published after the 2008 guidance for assessing harms in systematic reviews published by the Agency for Healthcare Research and Quality (AHRQ),[22] including 36/70 (51%) published between 2016 and 2020. We identified reviews for all the conditions/indications that we anticipated. Most reviews evaluated gabapentin as a single intervention by combining doses across and within studies (61/70; 87%). Eleven (16%) reviews included network meta-analysis (one of which also included pairwise meta-analysis). Of the 60 reviews making direct pairwise comparisons with gabapentin, placebo was the most common comparator, used in 52 (87%) reviews (Table 1A). Three quarters of reviews (51/70, 73%) reported following a guideline for systematic review methods or reporting, commonly PRISMA (26/70, 37%) and Cochrane (23/70, 33%). Seventeen (24%) reviews were not funded and 12/70 (17%) did not report a source of funding. The most commonly reported source of funding was government (24/70, 34%); one review was funded by a pharmaceutical manufacturer (Table 1A).

### 3.3. Methods for assessing harms: approach/planning

No reviews stated they followed any guidance for synthesizing harms (Table 2A).

Most reviews (60/70, 86%) aimed to assess the evidence for both potential benefits and harms—often with a greater focus on benefit—while 10/70 (14%) reviews focused solely on assessing harms. A hybrid approach—wherein at least one pre-specified harm was addressed alongside additional harms identified during the review—was taken by 18 of 70 (26%) reviews. Nearly equal numbers of reviews either did not pre-specify any harms of interest (27/70, 39%) or assessed only pre-specified harms (25/70, 36%) (Table 2A). Among the 28 reviews for which a protocol was available, either through publication or registration (e.g., PROSPERO), harms were mentioned and addressed in 24 (86%) (Table 2A).

Reviews often assessed multiple types of harms, most commonly specific ‘unique harms’ such as dizziness, somnolence, and weight gain (56/70, 86%). Reviews also commonly assessed harms using proxies such as “drop-out” or “loss-to-follow-up due to harms” (35/70, 50%) (Table 2a).

### 3.4. Methods for assessing harms: searching

Only 22/70 (31%) reviews performed supplemental searches for unpublished studies or data from regulatory agencies or industry. Table 1B presents characteristics of the searches used by the included reviews. The median [interquartile range] number of databases searched was 4 [3 to 5]. Most reviews had multiple search components, including: searching references, contacting experts in the field, searching for grey literature or conference abstracts, or searching registries for ongoing studies. Almost all reviews included randomized controlled trials (69/70, 99%); 43 (61%) included no other study types.

We found that not many reviews searched for types of studies used only to assess harms (e.g., observational studies of harms) (Table 1B). Our subgroup analyses based on the review purpose (i.e., assessing harms or benefits) and the approach to pre-specification of harms (i.e., whether any harms were pre-specified) found no systematic differences in the search methods or the types of evidence included in reviews. APPENDIX D contains the table of search methods and types of evidence by subgroup.

### 3.5. Methods for assessing harms: analyses

Of the 70 included reviews, 26 (37%) assessed harms only descriptively (i.e., did not use meta-analysis for any harms), 19 (27%) assessed all harms quantitatively, and 25 (36%) assessed harms using both descriptive and quantitative methods (Table 2B). Where quantitative estimates for harms were reported, relative measures were used more often (e.g., risk ratio, odds ratio) than absolute measures for harms (e.g., risk difference) (Table 2B).

Of the 70 reviews, 44 (63%) performed a quantitative analysis of at least one harm. Eleven (25%) of the 44 conducted a network meta-analysis for harm or a proxy for harm (Table 2B). Only three reviews specified their analysis methods were different for harms than benefit outcomes in either a protocol or a final report—all others that included a meta-analysis (41/44, 93%) appeared to use the same analysis methods for both benefit and harm outcomes. The method used to handle rare events in meta-analysis (i.e., including zero-event cells from studies) can affect the validity of the estimates[35,36] and we found including studies with zero-events in one treatment group was done in a greater number of reviews than including studies with no events in either the gabapentin or comparison group (Table 2B). Most of the reviews with a quantitative analysis 80% (35/44) did not report any approach to handling missing data from included studies (e.g., imputing missing standard deviations of estimates) (Table 2B).

Most reviews specified whether they used fixed-effect or random-effects models used for analysis. Only three (7%) reviews did not report what type of meta-analysis they conducted for harms: 32/44 (73%) reported at least one random-effects analysis and 15/44 (34%) reported at least one fixed-effects analysis for harms. Although the defaults for meta-analysis in most statistical programs used to conduct meta-analysis (e.g., RevMan, R, Stata) are inverse-variance models, which are often biased for analyzing rare events,[36–39] we found the most common meta-analysis model was Mantel-Haenszel (19/44, 43%) with only 9/44 (21%) reviews not specifying what model was used for meta-analysis, suggesting most systematic reviewers know to change the model from the default when analyzing harms.

### 3.6. Methods for assessing harms: reporting

Harms reporting was often unclear and inconsistent. Most reviews (57/70, 81%) included a statement about harms in the abstract; either about specific harms (31/57, 54%) or a more general statement about the potential for harm (26/57, 46%) (Table 2A). Many reviews (31/70; 44%) did not state whether any selection criteria had been used in the reporting of harms, and 37% (26/70) reported only pre-specified harms (Table 2A). Selection criteria are the rules defining which harms will be reported; these often involve numerical thresholds (e.g., “harms occuring in at least 2% of participants”) (Box 1). Selection criteria were used in 13/70 (19%) reviews, including both vague criteria such as reporting only “the most common harms” and more specific criteria such as reporting “only harms commonly reported in all included studies”.

Most reviews (46/70, 66%) included a statement about limitations specifically for harms. These limitations were commonly about adverse effects being poorly or inconsistently reported among included studies, trial study designs (e.g., sample size, duration, etc.) being insufficient for identifying harms, limiting included studies to trials, and not searching uncontrolled or unpublished literature.

## 4. Discussion

The desire to address harms in systematic reviews has led to tokenism. Guidance indicates that all reviews should assess harms,[2,7,22] but reviews rarely focus on harms, and reviews focusing on benefits rarely use appropriate methods to identify and synthesize evidence about harms. Some limitations in reviews stem from limitations in the included studies that could not be corrected with improved systematic review methods. Other limitations in the reviews stem from the methods chosen by reviewers, including previously identified limitations that have not yet been addressed.[4,6,15,20,21,40–42] Additionally, with a focus on potential benefits, most intervention reviews are limited to randomized controlled trials.[2,6] Randomized controlled trials are rarely designed to address harms, and publicly available evidence about trials is usually insufficient for assessing harms.[2,18,19,41–47] Consequently, systematic reviews of trials may be doomed to draw premature and sometimes invalid conclusions about the balance of benefits and harms.[3,6,22,48–51]

To address these challenges to synthesizing information about harms, a paradigm shift is needed. Cochrane and other producers of systematic reviews should reconsider how best to guide review authors to assess harms in systematic reviews that are designed primarily to assess potential benefits. We believe that systematic reviews specifically focused on harms are needed. Because many drugs are used for multiple indications, systematic reviews of harms that are limited to specific indications will lead to incomplete evidence. When appropriate, Consciously separating reviews of benefits and reviews of harms could increase validity, avoid unintended overlap, and reduce overall burden on systematic reviewers.

Appropriate methods to review harms differ from appropriate methods to assess potential benefits. For example, multiple study types should be considered when answering questions about harms, so systematic reviewers should not exclude observational data at the outset of the review process.[3,7,22] While randomized controlled trials can assess harms, especially common harms, most are not designed to do so. Some, but not all, limitations could be overcome in studies with larger samples, more diverse participants, and longer duration. By comparison, non-randomized studies can be misleading because of uncontrolled biases and confounding, so reviewers interested in harms should have training and experience assessing both randomized and non-randomized studies. For example, whereas non-randomized studies of statins showed an increased risk of myalgias, randomized trials and meta-analyses of them have shown no increase in risk.[52] Additionally, reviewers should anticipate including unpublished data when published data on harms is incomplete or likely to be inadequate for addressing the review question.[13,14,41–46,51,53]

Second, the reporting of analyses of harms requires greater detail in reviews. Reviewers should specify how the analyses are conducted (whether they were assessed the same or differently from benefits) and key assumptions such as handling of rare events and missing data.[21,40] Common meta-analysis models for efficacy outcomes, and common assumptions for how to handle rare events and missing data from included studies, are problematic for rare events.[9,35–37,39] Thus, reviews about harms might require more statistical expertise and support compared with reviews about benefits.

Third, and perhaps most importantly, reviewers should discuss the choice of selection criteria for assessing and reporting harms (Box 1). Selection criteria lead to reporting bias in primary studies, and the additional application of selection criteria in systematic reviews further obfuscates the evidence on harms.[17–19,54] Moreover, it is often unclear whether and which selection criteria have been used Existing guidance for systematic reviews, such as the PRISMA-harms extension, does not address the use of selection criteria.[8] When selection criteria are not reported, users of systematic reviews will not know if reported harms include: (a) all harms identified in all included studies; (b) only harms that were pre-specified; or (c) a subset of all harms selected post hoc. Part 2 of this methodologic study looks more closely at the results for harms of these reviews.

Reviews of harms require resources and information that are unavailable to many academic researchers. In this regard, responsibility for conducting and publishing systematic reviews of harms may fall on government organizations. For pharmaceuticals and devices, regulators such as the US Food and Drug Administration are well-positioned to conduct these reviews properly because they have access to individual participant data from trials, and they have epidemiologists and biostatisticians skilled at analyzing real world data on harms. Although regulators might be unable to undertake all the research they would like to conduct, publishing studies they do conduct would be valuable contribution to policy and practice.

Lastly, reviews should describe the limitations of any harms assessments. Following every recommendation above might be impossible or unnecessary in every review. so if recommended methods are not followed, then reviewers should provide a rationale and report any associated limitations so that readers can evaluate their confidence in the results. Peer reviewers and journal editors should strive to ensure that reporting guidelines are followed and that systematic reviews do not present overly confident summaries of harms when they use unreliable methods.

## 5. Conclusions

Consistent with the results of previous research, the current methods of assessing harms in the systematic reviews we examined does not produce valid evidence about harms. The underlying issues we identified demand a paradigm shift rather than incremental change or further guidance. Harms are currently included as tokenistic outcomes in reviews for the sake of assessing the “balance” of benefits and harms. We propose that reviewers fundamentally revise how we approach harms from review conception through resource collection and analysis to reporting.

## Supplementary Material

4

3

2

1

## Figures and Tables

**Table 1A.** Characteristics of included reviews of gabapentin (n = 70)

Review Characteristic	n	(%)
Year published		
2001–2005	4	(6%)
2006–2010	11	(16%)
2011–2015	19	(27%)
2016–2020	36	(51%)
Review condition(s)/indication(s) ^i^		
Neuropathic pain (e.g., phantom limb-pain, neuropathic cancer-pain)	18	(25%)
Post-operative pain	18	(25%)
Epilepsy (e.g., essential tremor, seizure disorders)	12	(17%)
Postherpetic neuralgia	7	(10%)
Fibromyalgia	6	(9%)
Migraine headaches	5	(7%)
Vasomotor symptoms (i.e., hot flashes)	4	(6%)
Alcohol dependence	3	(4%)
Psychiatric disorders (bipolar disorder, attention deficit disorder, obsessive compulsive disorder, post-traumatic stress disorder)	1	(1%)
Restless leg syndrome	1	(1%)
Non-specific–review focused on specific drugs	7	(10%)
Gabapentin assessed as …		
Single intervention (with one or more doses)	61	(87%)
Multiple interventions (separated by dose)	9	(13%)
Comparators evaluated against gabapentin ^ii^		
Direct comparisons against gabapentin (n = 106)	60	(86%)
Placebo	52	
Antiepileptic drugs	11	
Tricyclic antidepressants	8	
Pregabalin	4	
NSAIDs	4	
SNRIs or SSRIs	3	
Benzodiazepines	2	
Opioids	3	
Other (cannabinoids, topical capsaicin/lidocaine, vitamin E, estrogen, isoflavones, tibolone, hypnotic induction, electroacupuncture, etc.)	17	
Network Meta-Analysis (all interventions compared with each other)	11	(16%)
Specific guidance followed for general review methods/reporting ^iii^		
PRISMA	26	(37%)
Cochrane	23	(33%)
AHRQ	2	(3%)
FDA	0	(0%)
IOM	0	(0%)
Other (e.g., BMJ Clinical Evidence, NHS R&D HTA, ISPOR, PROSPECT)	9	(13%)
Source of funding or material support ^iv^		
Government (e.g., National Institutes of Health)	24	(34%)
Foundation	12	(17%)
Department, institution, or organization	11	(16%)
Pharmaceutical industry	1	(1%)
Explicit statement that there was no funding	17	(24%)
Not reported	12	(17%)

i Reviews could assess multiple conditions/indications

ii Reviews could include both direct, pairwise, comparisons and network meta-analyses

iii Reviews could report following multiple different guidance documents

iv Reviews could report multiple different sources of funding

**Table 1B.** Characteristics of searches and included studies from reliable systematic reviews of gabapentin (n = 70)

Review Characteristic	n		(%)	
Specific databases searched				
PubMed/MEDLINE	70		(100%)	
Cochrane	63		(90%)	
Embase	57		(81%)	
Psych INFO	14		(20%)	
CINAHL	12		(17%)	
Other^i^	38		(54%)	
Total number of bibliographic databases searched (median [IQR])	4 [3 to 5]			
Additional primary searching beyond bibliographic databases				
Reference lists of included reports	60		(86%)	
Experts in the field or included study authors	35		(50%)	
Unpublished/difficult to access literature (e.g., Grey literature, company reports, FDA data, conference abstracts)	39		(56%)	
Ongoing studies (e.g., clinical trial registries)	34		(49%)	
Types of included studies	*For general review*		*Specifically for harms*	
Randomized controlled trial	69	(99%)	0	(0%)
Controlled clinical trial	12	(17%)	4	(6%)
Cohort study	5	(7%)	4	(6%)
Case-control study	3	(4%)	5	(7%)
Case series/report	4	(6%)	2	(3%)
Reviews	13	(19%)	0	(0%)
Surveillance system	1	(1%)	6	(9%)
Other (e.g., Registry studies, cross-sectional studies, medical chart reviews, “noncomparative observational studies with duration ≥ 1 year”)	4	(6%)	2	(3%)
Supplemental searching for data (*beyond bibliographic databases, registries, references, and experts*)				
Unpublished studies or data	22	(31%)	3	(4%)
AE reporting systems	0	(0%)	5	(7%)
Hospital or other databases	0	(0%)	1	(1%)
Median [IQR] # studies (total) | participants (total) ^ii^	34.5 [14 to 78] | 3355 [1204 to 9181]			
Median [IQR] # studies (gabapentin) | participants (gabapentin) ^iii^	6 [3 to 16] | 420.5 [185 to 1114]			

i “Other” databases include (alphabetically): Allied and Complementary Medicine Database; Chinese Biological Medical Literature; China National Knowledge Infrastructure; Google Scholar; International Pharmaceutical Abstracts; Latin American and Caribbean Health Sciences Literature; Manual, Alternative and Natural Therapy Index System; National Institute for Health and Care Excellence; NHS databases (Health Economic Evaluation, Centres for Reviews and Dissemination Database of Abstracts of Reviews of Effects); Oxford Pain Relief Database; Pharmline; Reprotox; Scopus; Turning Research Into Practice; Wanfang Data; Web of Science/Science Citation Index

ii Participant median and IQR derived from 50 reviews that reported the total number of participants

iii Participant median and IQR derived from 34 reviews that reported the number of gabapentin participants

**Table 2A.** Methods for approaching and reporting harms among 70 reliable systematic reviews of gabapentin

Harms assessment	n	(%)
Approach to assessing harms		
Exploratory only	27	(39%)
Pre-specification only	25	(36%)
Hybrid (*≥* 1 pre-specified)	18	(26%)
Specific guidance followed for assessing harms		
Yes	0	(0%)
Not reported	70	(100%)
Types of harms analyzed in reviews^i^		
Separate and specific harms	56	(80%)
Drop-out due to harms	35	(50%)
Any non-specific harms	29	(41%)
Grouped specific harms	9	(13%)
Other (e.g., serious adverse events)	9	(13%)
Reporting of harms		
Review protocol addresses harms		
No protocol/registration mentioned in review	30	(43%)
Protocol/registration mentioned, but not available	12	(17%)
Protocol/registration obtained: NO, harms not addressed	4	(6%)
Protocol/registration obtained: YES, harms are addressed	24	(34%)
Abstract includes statement on harms		
Yes	57	(81%)
Specific statement	31	
General statement	26	
No	13	(19%)
Use of selection criteria		
Reported only pre-specified harms	26	(37%)
Yes, selection criteria: Reported a subset of harms identified	13	(19%)
Unclear–no statement on use of criteria	31	(44%)
Reported limitations of harms assessment	46	(66%)

i Reviews could assess multiple types of harms

**Table 2B.** Methods for analysis of harms among 70 reliable systematic reviews of gabapentin

Analysis methods for harms^i^	n	(%)
Approach to assessing harms		
Both descriptive and quantitative	25	(36%)
Descriptive only	26	(37%)
Quantitative only (i.e., meta-analysis)	19	(27%)
Measure of effect ^ii^		
Risk Ratio	20	(29%)
Odds Ratio	12	(17%)
Count	10	(14%)
Number Needed to Harm	5	(7%)
Risk Difference	2	(3%)
Risk	2	(3%)
Mean increase	1	(1%)
Not reported (no estimate)	37	(53%)
Quantitative analysis methods	(n = 44)	
Reported different analysis methods specifically for harms		
Yes	3	(7%)
No	41	(93%)
Inclusion of zero-event studies in meta-analysis		
Yes	9	(20%)
No	4	(9%)
Not reported	31	(71%)
Inclusion of zero-cell studies in meta-analysis		
Yes	18	(41%)
Not reported	26	(59%)
Type of analysis		
Fixed effects	15	(34%)
Random effects	32	(73%)
Not reported	3	(7%)
Analysis model		
Mantel-Haenszel	19	(43%)
Dersimonian and Laird	5	(11%)
Inverse variance	3	(7%)
Peto-OR	1	(2%)
Network meta-analysis (Bayesian or frequentist)	11	(25%)
Not reported	8	(18%)
Handling of missing data		
Simple imputation	4	(9%)
Last value carried forward	3	(7%)
Sensitivity analysis	2	(5%)
Multiple imputation	1	(2%)
Ignore (i.e., complete case)	1	(2%)
Not reported	35	(80%)

i None of the analysis elements are mutually exclusive as reviews may take different approaches for multiple harms, from assessing some descriptively and others quantitatively, to using different quantitative models for different harms

ii At least one harm included in the review had an overall summary estimate with ___ measure of effect.

## What is new?

- Despite the existence of guidelines and recommendations for how to assess harms in systematic reviews, reviews we examined do not appear to adhere to best practices.
- Synthesizing evidence of harms is more challenging and requires substantially more effort than assessing benefits; however most reviews examined in this study had methods oriented toward answering questions of benefit.
- Selection criteria applied to harms in primary studies affect the completeness of harms data, yet reviews also have selection criteria that further bias their results and limit information available to evidence users.
- The current approach to assessing harms in systematic reviews needs immediate improvement.

## Box 1 Glossary of terms related to synthesis of harms

**Table:**

Term	Definition
Terms related to harms	
Harms	“Harms” is a general umbrella term to cover the concept of risk that may be associated with an intervention. “Harms” is used to refer to all related ideas, such as adverse events, side effects, tolerability, or safety.
Systematically collected harms	According to The Final Rule, ‘‘‘systematic assessment’ involves the use of a specific method of ascertaining the presence of an adverse event (e.g., the use of checklists, questionnaires, specific laboratory tests at regular intervals)’’. Like a potential benefit of treatment, a systematic AE can be defined using five elements: (1) domain, (2) specific measurement, (3) specific metric, (4) method of aggregation, and (5) time-point [13]. For example, ‘‘proportion of participants with 50% change from baseline to 8 weeks on the Young Mania Rating Scale total score.’’
Non-systematically collected harms	According to The Final Rule, ‘‘‘non-systematic assessment’ relies on the spontaneous reporting of adverse events, such as unprompted self-reporting by participants.’’ Non-systematic adverse events may be collected by asking questions like ‘‘Have you noticed any symptoms since your last examination?’’
Unique harms	A specific harm such as would be reported by someone receiving an intervention, such as “dizziness”, “edema”, or “somnolence”.
General assessment of harm	A non-specific method of assessing harms that aims to summarize multiple aspects of risk into a single measure, such as “occurrence of any harm”, “occurrence of serious adverse events”, or a composite of several unique harms.
Proxy for harm	A surrogate method of assessing harm that is not a direct representation of harm from an intervention, such as “loss-to-follow-up or drop-out due to harms”.
Terms related to review methods	
Pre-specification of harms	An approach to assessing harms in systematic reviews whereby reviewers have one or more harms in mind that they consider important and pre-specify as outcomes of interest for their review. These pre-specified harms are the only harms that are assessed in the review.
No pre-specification of harms (“exploratory”)	An approach to assessing harms in systematic reviews whereby reviewers do not pre-specify any harms of interest as outcomes for their review. Reviewers assess only harms identified in the review process. A review can specify that they will broadly assess harms as an outcome and still be exploratory if they do not note any specific harms of interest.
Hybrid	An approach to assessing harms in systematic reviews whereby reviewers pre-specify at least one harm to assess in the review and also assess harms identified during the review process.
Primary search for evidence	The principal sources of evidence for a search that are recommended as standard for all systematic reviews, including: bibliographic databases, grey literature, study registries, content experts, and reference lists of included studies.
Supplemental search for evidence	The additional sources of evidence for a search that are not standard for systematic reviews and are uncommonly performed, including: searching unpublished data, adverse event reporting systems, and hospital and other databases.
Descriptive assessment of harm	A narrative description of the harm(s) reported in studies included in the review that does not involve meta-analysis of estimates across studies.
Quantitative assessment of harm	The statistical combination of estimates for harm(s) across two or more studies included in the review. (i.e., meta-analysis for a harm).
Selection criteria	The specific rules that are used to define a subset of harms that will be reported among all of the harms collected. Selection criteria are often based on numerical threshold and participant group (e.g., ≥ 5% of participants in the intervention group).

## Box 2 Guidance for synthesizing harms

### Approach

- Assess multiple harms and focus on specific harms (as opposed to general assessments of harm);
- Sources of evidence
- Search multiple sources of evidence to identify information about harms;
- Utilize any unpublished data for included studies that can be obtained;
- For reviews that include harms, do not restrict studies to randomized controlled trials; instead, include observational studies, case reports, adverse event reporting systems;

### Methods of analysis

- Absolute measures (e.g., risk difference) might give a better indication of public health impact over relative measures (e.g., risk ratio and odds ratio) when conducting meta-analyses, although these are complementary and should both be considered;
- Avoid inverse-variance and Dersimonian and Laird methods when selecting a model for meta-analysis for rare events, and give preference to Bayesian or select frequentist models (Peto one-step odds ratio or Mantel-Haenszel odds ratio without zero-cell corrections);

### Reporting

- Report methods for assessing harms in systematic reviews, including details of how the primary studies assessed harms.

## References

[1] Cochrane Collaboration Cochrane Handbook for Systematic Reviews of Interventions Higgins J, Green S, editors. Wiley; 2019. doi:10.1002/9780470712184.ch5.

[2] Thomas J, Kneale D, Mckenzie JE, Brennan SE, Bhaumik S. Chapter 2: Determining the scope of the review and the questions it will address. Cochrane Handbook for Systematic Reviews of Interventions. 6.0; 2019.

[3] Peryer G, Golder S, Junqueira D, Vohra S, Kong Loke Y, Chapter 19: Adverse effects Cochrane Handbook for Systematic Reviews of Interventions. Version 6. Cochrane Higgins J, Thomas J, Chandler J, et al., editors; 2019 https://training.cochrane.org/handbook/version-6/chapter-19-draftv2.

[4] Ernst E, Pittler MH. Assessment of therapeutic safety in systematic reviews: literature review. Br Med J 2001;323(7312):546. doi:10.1136/bmj.323.7312.546. 11546700 PMC48159

[5] Etminan M, Carleton B, Rochon PA. Quantifying adverse drug events: Are systematic reviews the answer? Drug Saf 2004;27(11):757–61. 15350149 10.2165/00002018-200427110-00001

[6] McIntosh HM, Woolacott NF, Bagnall AM. Assessing harmful effects in systematic reviews. BMC Med Res Methodol 2004;4(August). doi:10.1186/1471-2288-4-19. PMC497041 15260887

[7] Committee on Standards for Systematic Reviews of Comparative Effectiveness Research; Institute of Medicine of the National Academies Finding What Works in Healthcare: Standards for Systematic Reviews Eden J, Levit L, Berg A, Morton S, editors. The National Academies Press; 2011. doi:10.1016/b0-32-300162-9/50007-6. 24983062

[8] Zorzela L, Loke YK, Ioannidis JPA, PRISMA Harms: improving harms reporting in systematic reviews. Br Med J 2016;352(157):1–17. doi:10.1136/bmj.i157. 26830668

[9] Center for Drug Evaluation and Research. Meta-Analyses of Randomized Controlled Clinical Trials to Evaluate the Safety of Human Drugs or Biological Products; 2018.

[10] Bennetts M, Whalen E, Ahadieh S, Cappelleri JC. An appraisal of meta-analysis guidelines: How do they relate to safety outcomes? Res Synth Methods 2017;8(1):64–78. doi:10.1002/jrsm.1219. 27612447

[11] Dwan K, Altman DG, Arnaiz JA, Systematic review of the empirical evidence of study publication bias and outcome reporting bias. PLoS One 2008;3(8):e3081. doi:10.1371/journal.pone.0003081. 18769481 PMC2518111

[12] Hopewell S, Wolfenden L, Clarke M. Reporting of adverse events in systematic reviews can be improved: Survey results. J Clin Epidemiol 2008;61(6):597–602. doi:10.1016/j.jclinepi.2007.10.005. 18411039

[13] Kirkham JJ, Dwan KM, Altman DG, The impact of outcome reporting bias in randomised controlled trials on a cohort of systematic reviews. Br Med J 2010;340:c365. doi:10.1136/bmj.c365. 20156912

[14] Dwan K, Gamble C, Williamson PR, Kirkham JJReporting Bias Group. Systematic review of the empirical evidence of study publication bias and outcome reporting bias - An updated review. PLoS One 2013;8(7):e66844. doi:10.1371/journal.pone.0066844. 23861749 PMC3702538

[15] Saini P, Loke YK, Gamble C, Altman DG, Williamson PR, Kirkham JJ. Selective reporting bias of harm outcomes within studies: Findings from a cohort of systematic reviews. Br Med J 2014;349:g6501. doi:10.1136/bmj.g6501. 25416499 PMC4240443

[16] Zorzela L, Golder S, Liu Y, Quality of reporting in systematic reviews of adverse events: Systematic review. Br Med J 2014;348:f7668. doi:10.1136/bmj.f7668. 24401468 PMC3898583

[17] Mayo-Wilson E, Fusco N, Li T, Hong H, Canner JK, Dickersin K. Multiple outcomes and analyses in clinical trials create challenges for interpretation and research synthesis. J Clin Epidemiol 2017;86:39–50. doi:10.1016/j.jclinepi.2017.05.007. 28529187

[18] Mayo-Wilson E, Fusco N, Li T, Hong H, Canner JK, Dickersin K. Harms are assessed inconsistently and reported inadequately Part 1: Systematic adverse events. J Clin Epidemiol 2019;113:20–7. doi:10.1016/j.jclinepi.2019.04.022. 31055175

[19] Mayo-Wilson E, Fusco N, Li T, Hong H, Canner JK, Dickersin K. Harms are assessed inconsistently and reported inadequately Part 2: Non-systematic adverse events. J Clin Epidemiol 2019;113:11–19. doi:10.1016/j.jclinepi.2019.04.020. 31055176

[20] Golder S, Loke Y, McIntosh HM. Poor reporting and inadequate searches were apparent in systematic reviews of adverse effects. J Clin Epidemiol 2008;61(5):440–8. doi:10.1016/j.jclinepi.2007.06.005. 18394536

[21] Cornelius V, Perrio M, Shakir SA, Smith L. Systematic reviews of adverse effects of drug interventions: a survey of their conduct and reporting quality. Pharmacoepidemiol Drug Saf 2009;18(September):1223–31. doi:10.1002/pds.1844. 19757414

[22] Chou R, Aronson N, Atkins D, Assessing Harms When Comparing Medical Interventions: Methods Reference Guide for Effectiveness and Comparative Effectiveness Reviews; 2008. doi:10.1016/j.jclinepi.2008.06.007

[23] Qureshi R, Mayo-Wilson E, Li T. Summaries of harms in systematic reviews are unreliable Paper 1: An introduction to research on harms. J Clin Epidemiol 2021 (IN PRESS). 10.1016/j.jclinepi.2021.10.023 PMC9126149 34742788

[24] Qureshi R, Mayo-Wilson E, Rittiphairoj T, McAdams-DeMarco M, Guallar E, Li T. Summaries of harms in systematic reviews are unreliable Paper 3: Given the same data sources, systematic reviews of gabapentin have different results for harms. J Clin Epidemiol 2021 (IN PRESS). 10.1016/j.jclinepi.2021.10.025 PMC9875741 34742790

[25] Peckham AM, Evoy KE, Ochs L, Covvey JR. Gabapentin for off-label use: evidence-based or cause for concern? Subst Abus Res Treat 2018;12. doi:10.1177/1178221818801311. PMC6153543 30262984

[26] Page MJ, McKenzie JE, Bossuyt PM, The PRISMA 2020 statement: An updated guideline for reporting systematic reviews. Syst Rev 2021;10(1). doi:10.1186/s13643-021-01626-4. PMC8008539 33781348

[27] Golozar A, Chen Y, Lindsley K, Identification and description of reliable evidence for 2016 American academy of ophthalmology preferred practice pattern guidelines for cataract in the adult eye. JAMA Ophthalmol 2018;136(5):514–23. doi:10.1001/jamaophthalmol.2018.0786. 29800249 PMC6145658

[28] Mayo-Wilson E, Ng SM, Chuck RS, Li T. The quality of systematic reviews about interventions for refractive error can be improved: A review of systematic reviews. BMC Ophthalmol 2017;17:164. doi:10.1186/s12886-017-0561-9. 28870179 PMC5584039

[29] Le JT, Qureshi R, Twose C, Evaluation of systematic reviews of interventions for retina and vitreous conditions. JAMA Ophthalmol 2019;137(12):1399–406. doi:10.1001/jamaophthalmol.2019.4016. 31600387 PMC6802257

[30] Yu T, Li T, Lee K, Friedman D, Dickersin K, Puhan M. Setting priorities for comparative effectiveness research on management on primary angle closure: A survey of Asia-Pacific clinicians. J Glaucoma 2015;24(5):348–55. doi:10.1016/j.physbeh.2017.03.040. 23835674 PMC3883875

[31] Lindsley K, Li T, Ssemanda E, Virgili G, Dickersin K. Interventions for age-related macular degeneration: Are practice guidelines based on systematic reviews? Ophthalmology 2016;123(4):884–97. doi:10.1016/j.physbeh.2017.03.040. 26804762 PMC4808456

[32] Li T, Vedula S, Scherer R, Dickersin K. What comparative effectiveness research is needed? A framework for using guidelines and systematic reviews to identify evidence gaps and research priorities. Ann Intern Med 2012;156(5):367–377. doi:10.1038/jid.2014.371. 22393132 PMC3804310

[33] Li T, Saldanha IJ, Jap J, A randomized trial provided new evidence on the accuracy and efficiency of traditional vs. electronically annotated abstraction approaches in systematic reviews. J Clin Epidemiol 2019;115:77–89. doi:10.1016/j.jclinepi.2019.07.005. 31302205

[34] Agency for Healthcare Research and Quality. Systematic Review Data Repository US Department of Health and Human Services. Published 2020. https://srdr.ahrq.gov

[35] Weber F, Knapp G, Ickstadt K, Kundt G, Glass Ä. Zero-cell corrections in random-effects meta-analyses. Res Synth Methods 2020(September):1–7. doi:10.1002/jrsm.1460. 32991790

[36] Sweeting MJ, Sutton AJ, Lambert PC. What to add to nothing? Use and avoidance of continuity corrections in meta-analysis of sparse data. Stat Med 2004;23(9):1351–75. doi:10.1002/sim.1761. 15116347

[37] Deeks JJ, Higgins JP, Altman DG, Chapter 10: Analysing data and undertaking meta-analyses. In: Higgins J, Thomas J, Chandler J, et al., editors. Cochrane Handbook for Systematic Reviews of Interventions. Version 6. Cochrane; 2019. p. 241–84. doi:10.1002/9781119536604.ch10.

[38] Efthimiou O Practical guide to the meta-analysis of rare events. Evid Based Ment Health 2018;21(2):72–6. doi:10.1136/eb-2018-102911. 29650528 PMC10270432

[39] Bradburn MJ, Deeks JJ, Berlin JA, Localio AR. Much ado about nothing: A comparison of the performance of meta-analytical methods with rare events. Stat Med 2007;26:53–77. doi:10.1002/sim.2528. 16596572

[40] Golder S, Loke Y, McIntosh HM. Room for improvement? A survey of the methods used in systematic reviews of adverse effects. BMC Med Res Methodol 2006;6:2–7. doi:10.1186/1471-2288-6-3. 16441876 PMC1402311

[41] Golder S, Loke YK, Wright K, Norman G. Reporting of adverse events in published and unpublished studies of health care interventions: A systematic review. PLoS Med 2016;13(9):e1002127. doi:10.1371/journal.pmed.1002127. 27649528 PMC5029817

[42] Golder S, Loke YK, Wright K, Sterrantino C. Most systematic reviews of adverse effects did not include unpublished data. J Clin Epidemiol 2016;77:125–33. doi:10.1016/j.jclinepi.2016.05.003. 27259470

[43] Wieseler B, Wolfram N, McGauran N, Completeness of reporting of patient-relevant clinical trial outcomes: comparison of unpublished clinical study reports with publicly available data. PLoS Med 2013;10(10):e1001526. doi:10.1371/journal.pmed.1001526. 24115912 PMC3793003

[44] Golder S, Loke YK, Bland M. Unpublished data can be of value in systematic reviews of adverse effects: Methodological overview. J Clin Epidemiol 2010;63(10):1071–81. doi:10.1016/j.jclinepi.2010.02.009. 20457510

[45] Vedula SS, Bero L, Scherer RW, Dickersin K. Outcome reporting in industry-sponsored trials of gabapentin for off-label use. N Engl J Med 2009;361(20):1963–71. doi:10.1056/NEJMsa0906126. 19907043

[46] Vedula SS, Li T, Dickersin K. Differences in reporting of analyses in internal company documents versus published trial reports: Comparisons in industry-sponsored trials in off-label uses of gabapentin. PLoS Med 2013;10(1):e1001378. doi:10.1371/journal.pmed.1001378. 23382656 PMC3558476

[47] Mayo-Wilson E, Fusco N, Hong H, Li T, Canner JK, Dickersin K. Opportunities for selective reporting of harms in randomized clinical trials: Selection criteria for non-systematic adverse events. Trials 2019;20(1):553. doi:10.1186/s13063-019-3581-3. 31488200 PMC6728982

[48] Lamberti MJ, Kubick W, Awatin J, McCormick J, Carroll J, Getz K. The use of real-world evidence and data in clinical research and postapproval safety studies. Ther Innov Regul Sci 2018;52(6):778–83. doi:10.1177/2168479018764662. 29714579

[49] Papanikolaou PN, Christidi GD, Ioannidis JP. Comparison of evidence on harms of medical interventions in randomized and non-randomized studies. J Can Med Assoc 2006;174(5):635–41. doi:10.1503/cmaj.050873. PMC1389826 16505459

[50] Tsang R, Colley L, Lynd LD. Inadequate statistical power to detect clinically significant differences in adverse event rates in randomized controlled trials. J Clin Epidemiol 2009;62:609–16. doi:10.1016/j.jclinepi.2008.08.005. 19013761

[51] Doshi P, Jones M, Jefferson T. Rethinking credible evidence synthesis. Br Med J (Clin Res Ed) 2012;344(January):1–8. doi:10.1136/bmj.d7898. 22252039

[52] Peto R, Collins R. Trust the blinded randomized evidence that statin therapy rarely causes symptomatic side effects. Circulation 2018;138(15):1499–501. doi:10.1161/CIRCULATIONAHA.118.036846. 30354509

[53] Doshi P, Jefferson T, del Mar C. The imperative to share clinical study reports: Recommendations from the Tamiflu experience. PLoS Med 2012;9(4):e1001201. doi:10.1371/journal.pmed.1001201. 22505850 PMC3323511

[54] Mayo-Wilson E, Li T, Fusco N, Cherry-picking by trialists and meta-analysts can drive conclusions about intervention efficacy. J Clin Epidemiol 2017;91:95–110. doi:10.1016/j.jclinepi.2017.07.014. 28842290

