# Supplementary material for: Harms in Systematic Reviews Paper 2: Methods used to assess harms are neglected in systematic reviews of gabapentin
Source: J Clin Epidemiol. Author manuscript; Available in PMC 2023 Mar 1. (PMC9875742; doi:10.1016/j.jclinepi.2021.10.024)
Supplement: 4 [file NIHMS1858687-supplement-4.docx]

**APPENDIX D – Subgroup analysis**

**Subgroup: By approach to harms – pre-specification of any harms vs. exploratory only**

|  | **Pre-specify ≥ 1 harm**  **(n = 43)** | | | | **Exploratory**  **(n = 27)** | | | |
| --- | --- | --- | --- | --- | --- | --- | --- | --- |
| **Review Characteristic** | **n** | | **(%)** | | **n** | | **(%)** | |
| **Additional primary searching beyond bibliographic databases** |  | |  | |  | |  | |
| Reference lists of included reports | 37 | | (86%) | | 23 | | (85%) | |
| Experts in the field or included study authors | 21 | | (49%) | | 14 | | (52%) | |
| Unpublished/difficult to access literature (e.g., Grey literature, company reports, FDA data, conference abstracts) | 23 | | (53%) | | 16 | | (60%) | |
| Ongoing studies (e.g., clinical trial registries) | 25 | | (58%) | | 9 | | (33%) | |
|  | *For general review* | | *Specifically for harms* | | *For general review* | | *Specifically for harms* | |
| **Types of included studies** |  |  |  |  |  |  |  |  |
| Randomized controlled trial | 42 | (98%) | 0 | (0%) | 27 | (100%) | 0 | (0%) |
| Controlled clinical trial | 5 | (12%) | 2 | (5%) | 7 | (26%) | 2 | (7%) |
| Cohort study | 4 | (9%) | 2 | (5%) | 1 | (4%) | 2 | (7%) |
| Case-control study | 3 | (7%) | 2 | (5%) | 0 | (0%) | 3 | (11%) |
| Case series/report | 2 | (5%) | 1 | (2%) | 2 | (7%) | 1 | (4%) |
| Reviews | 6 | (14%) | 0 | (0%) | 7 | (26%) | 0 | (0%) |
| Surveillance system | 1 | (2%) | 2 | (5%) | 0 | (0%) | 4 | (15%) |
| Other | 3 | (7%) | 0 | (0%) | 1 | (4%) | 2 | (7%) |
| **Supplemental searching for data (*beyond bibliographic databases, registries, references, and experts*)** |  |  |  |  |  |  |  |  |
| Unpublished studies or data | 16 | (37%) | 3 | (7%) | 6 | (22%) | 0 | (0%) |
| AE reporting systems | 0 | (0%) | 2 | (5%) | 0 | (0%) | 3 | (11%) |
| Hospital or other databases | 0 | (0%) | 0 | (0%) | 0 | (0%) | 1 | (4%) |

**Subgroup: By review purpose – safety only vs. efficacy/efficacy and safety**

|  | **Safety**  **(n = 10)** | | | | **Efficacy/Efficacy and safety**  **(n = 60)** | | | |
| --- | --- | --- | --- | --- | --- | --- | --- | --- |
| **Review Characteristic** | **n** | | **(%)** | | **n** | | **(%)** | |
| **Additional primary searching beyond bibliographic databases** |  | |  | |  | |  | |
| Reference lists of included reports | 8 | | (80%) | | 52 | | (87%) | |
| Experts in the field or included study authors | 3 | | (30%) | | 32 | | (53%) | |
| Unpublished/difficult to access literature (e.g., Grey literature, company reports, FDA data, conference abstracts) | 4 | | (40%) | | 35 | | (58%) | |
| Ongoing studies (e.g., clinical trial registries) | 4 | | (40%) | | 30 | | (50%) | |
|  | *For general review* | | *Specifically for harms* | | *For general review* | | *Specifically for harms* | |
| **Types of included studies** |  |  |  |  |  |  |  |  |
| Randomized controlled trial | 9 | (90%) | 0 | (0%) | 60 | (100%) | 0 | (0%) |
| Controlled clinical trial | 3 | (30%) | 0 | (0%) | 9 | (15%) | 4 | (7%) |
| Cohort study | 4 | (40%) | 0 | (0%) | 1 | (2%) | 4 | (7%) |
| Case-control study | 3 | (30%) | 0 | (0%) | 0 | (0%) | 4 | (7%) |
| Case series/report | 2 | (20%) | 0 | (0%) | 2 | (3%) | 2 | (3%) |
| Reviews | 3 | (30%) | 0 | (0%) | 10 | (17%) | 0 | (0%) |
| Surveillance system | 1 | (10%) | 0 | (0%) | 0 | (0%) | 6 | (10%) |
| Other | 3 | (30%) | 0 | (0%) | 1 | (2%) | 2 | (3%) |
| **Supplemental searching for data (*beyond bibliographic databases, registries, references, and experts*)** |  |  |  |  |  |  |  |  |
| Unpublished studies or data | 1 | (10%) | 2 | (20%) | 21 | (35%) | 1 | (2%) |
| AE reporting systems | 0 | (0%) | 0 | (0%) | 0 | (0%) | 4 | (7%) |
| Hospital or other databases | 0 | (0%) | 0 | (0%) | 0 | (0%) | 1 | (2%) |
