# Supplementary material for: Harms in Systematic Reviews Paper 2: Methods used to assess harms are neglected in systematic reviews of gabapentin
Source: J Clin Epidemiol. Author manuscript; Available in PMC 2023 Mar 1. (PMC9875742; doi:10.1016/j.jclinepi.2021.10.024)
Supplement: 3 [file NIHMS1858687-supplement-3.docx]

**APPENDIX C – Reliability Assessment for Systematic Reviews**

**Criteria for assessing the reliability of systematic reviews**

| **Criterion** | **Definition applied to systematic review reports** |
| --- | --- |
| Defined eligibility criteria | Described inclusion and/or exclusion criteria for eligible studies. |
| Conducted comprehensive literature search | Review authors (1) described an electronic search of two or more bibliographic databases; (2) used a search strategy comprising a mixture of controlled vocabulary and keywords; (3) reported using at least one other method of searching such as searching of conference abstracts; identified ongoing trials; complemented electronic searching by hand search methods (e.g., checking reference lists); and contacted included study authors or experts. |
| Assessed risk of bias of included studies | Used any method (e.g., scales, checklists, or domain-based evaluation) designed to assess methodologic rigor of included studies. |
| Used appropriate methods for meta-analysis | Used quantitative methods that (1) were appropriate for the study design analyzed (e.g., maintained the randomized nature of trials; used adjusted estimates from observational studies); (2) correctly computed the weight for included studies. |
| Observed concordance between review findings and conclusions | Authors’ reported conclusions were consistent with findings, provided a balanced consideration of benefits and harms, and did not favor a specific intervention if there was lack of evidence. |

**Reliability assessment form:**

Q1. What is the objective of the systematic review?

*Record VERBATIM the objective of the systematic review described in one of the following sections of the article using this order of priority: Abstract, Methods, Background, Other. If not reported, insert NR in the text box.*

Q2. What is the main conclusion of the systematic review?

*Record VERBATIM the main conclusion(s) of the systematic review described in one of the following sections of the article using this order of priority: Abstract, Methods, Background, Other. If not reported, insert NR in the text box.*

Q3. In your judgment, did the author(s) ask at least one clearly-focused research question?

*Check “Yes” if at least the population and test intervention are specified in the review (e.g., Selective laser trabeculectomy for primary open angle glaucoma).*

- Yes
- No
- Can’t tell

Q4a. Did the author(s) report having eligibility criteria for including/excluding primary studies in the systematic review?

*Check "Yes," "No" or "Can't tell." If "Yes" is selected, answer Q4b. If "NO" or "CAN'T TELL" are selected, SKIP to Q5a.*

- Yes
- No
- Can’t tell

Q4b. Did the authors(s) state that the eligibility criteria were pre-specified (e.g., authors referenced a protocol or PROSPERO registration, or stated that the eligibility criteria were pre-specified)?

*Select "Yes" for all Cochrane systematic reviews because Cochrane reviews require publication of a protocol.*

- Yes
- No
- Can’t tell

Q5a. Did the authors report searching any of the following bibliographic databases to identify relevant studies?

*Check "Yes," "No" or "Not reported" for each category. BIBLIOGRAPHIC DATABASE is defined as an organized digital collection of references to published literature which provides descriptive records of items such as journal articles, book (chapter)s, and conference proceedings. Only note bibliographic databases; clinical trial registries and hand-searching do not need to be listed here.*

**Source Yes No Can’t tell**

Q5a1. PubMed of MEDLINE O O O

Q5a2. Cochrane (e.g., CENTRAL) O O O

Q5a3. EMBASE O O O

Q5a4. LILACS O O O

Q5a5. Other, specify (Q5a6): O O O

Provide rows not specified in the list:

Q5b1. What is the total number of bibliographic databases searched?

*Please count the number of databases explicitly stated in the methods. Insert the number only. If not reported, insert NR in the text box.*

Q5b2. What is the latest date of searching any bibliographic database?

[Month] / [Date] / [Year]

Q5c. Did the authors report searching non-English-language studies for at least one of the above bibliographic database(s)?

- Yes
- No
- Can’t tell

Q5d. Did the authors report searching for all possible years for at least one of the above bibliographic database?

*Years of origin/coverage for commonly searched databases: PubMed (1966), Medline (1946), Embase (1947), CINAHL (1961), Web of Science/Science Citation Index (1900) Note: "All possible years" implies the years relevant to the research question. For example, if a new device was invented in 2001, it would not be necessary to search from database inception. "All possible years" would reasonably be searching all articles since 2001, or perhaps several years earlier to potentially capture early phase studies (e.g., 1995 - present).*

- Yes
- No
- Can’t tell

Q5e. Did the authors report searching reference lists or search for reports that cited included studies?

- Yes
- No
- Can’t tell

Q5f. Did the authors report contacting experts in the field and/or contacted study authors?

- Yes
- No
- Can’t tell

Q5g. Did the authors report searching for unpublished or difficult to access studies (e.g., grey literature, FDA data, internal company reports, conference abstracts)?

- Yes
- No
- Can’t tell

Q5h. Did the authors report searching for ongoing studies (e.g., clinicaltrials.gov)?

- Yes
- No
- Can’t tell

Q5i. Overall, in your judgment and with all the presented information (e.g. search terms, number of trials identified), do you think that the search for evidence was reasonably comprehensive?

*If "No" is selected, add a note of your rationale in the comments. (Note, depending on your browser, the "notes" and comment box may appear at the very top of your screen).*

- Yes
- No…
- Can’t tell

Q6. Did the author(s) report assessing the risk of bias or methodological quality using any method in individual studies?

*Check "Yes," "No," "Can't tell," or "Not applicable." Many methods exist to assess the risk of bias, including scales, in which various components of quality are scored and combined to give a summary score; or checklists, in which specific questions are asked; or domain-based evaluation, in which critical assessment are made separately for different domain, for example, allocation concealment, making etc. Select "Not applicable" if the review included zero studies.*

- Yes
- No
- Can’t tell
- Not applicable

Q7. Reviewers involved in data abstraction:

*Select one response in each column*

**Abstraction element Number Independently?**

Q7a. Assessed each title and/or abstract’s eligibility? O 1 O Yes

O 2 or more O No

O Not reported O Not reported

O Not applicable O Not applicable

Q7b. Assessed each full text article’s eligibility? O 1 O Yes

O 2 or more O No

O Not reported O Not reported

O Not applicable O Not applicable

Q7c. Assessed the risk of bias of each included study? O 1 O Yes

O 2 or more O No

O Not reported O Not reported

O Not applicable O Not applicable

Q7d. Abstracted data from each included study? O 1 O Yes

O 2 or more O No

O Not reported O Not reported

O Not applicable O Not applicable

Q8a. How did the author(s) combine the results?

*Qualitative synthesis involves the author(s) describing the characteristics and risk of bias (methodological quality) in individual studies that may affect the cumulative evidence. Skip to Q9a if you answer “no” or “Can’t tell” in Q8a2.*

**Analyses Yes No Can’t tell**

Q8a1. Qualitatively O O O

Q8a2. Quantitatively (meta-analysis) O O O

Q8b. Did the authors report statistical heterogeneity?

*STATISTICAL HETEROGENEITY - Used to describe the degree of variation in the effect estimates from a set of studies. Also used to indicate the presence of variability among studies beyond the amount expected due solely to the play of chance.*

- Yes
- No
- Can’t tell

Q8c. Given the clinical, design and statistical heterogeneity, was it reasonable to combine results in a meta-analysis?

- Yes
- No
- Can’t tell

Q8d. Were the methods used to combine the results of studies appropriate?

*Select "No" if incorrect variance or meta-analysis formula were used, and treatment effects were meta-analyzed by arms and therefore broke randomization.*

- Yes
- No
- Can’t tell

Q9a. Was source(s) of monetary or material support ("funding") for study reported in article?

*If "Yes" is selected, complete Q9b. IF "NO" IS SELECTED, SKIP to Q10.*

- Yes
- No

Q9b. What was the reported source(s) of monetary or material support?

*Select one response in each column*

**Funding Yes No**

Q9bi. Government (e.g., National Institutes of Health) O O

Q9bii. Pharmaceutical industry O O

Q9biii. Other industry O O

Q9biv. Foundation O O

Q9bv. Department, institution, or organization O O

Q9bvi. The authors explicitly reported there was no funding source O O

Q9bvii. Other, specify (Q9bviii): O O

Provide rows not specified in the list:

Q10. Did authors report any information on financial relationships?

*Note: We are asking about the authors of the systematic review, not the authors of the studies included in the systematic review.*

- Yes, authors reported explicitly that none of the authors has any financial relationships
- Yes, authors reported that there were some financial relationships
- No, authors did not report financial relationships

Q11. In your judgment, do you think the conclusions related to the main research question of the systematic review are supported by the data?

- Yes
- No
- Can’t tell

Q12a. Overall, in your judgment, do you think the systematic review is reliable?

*IF YES, SKIP Q12b: the assessment is complete. If "No" is selected, answer Q12b.*

- Yes
- No

Q12b. Please report the reason why the systematic review is unreliable?

*Check "Yes," "No" or "Not applicable" for each category.*

**Reason Yes No Not applicable**

Q12bi. Did not define eligibility criteria O O O

Q12bii. Did not conduct a comprehensive search O O O

Q12biii. Did not assess risk of bias of included studies O O O

Q12biv. Used inappropriate quantitative methods for meta-analysis O O O

Q12bv. Conclusions not supported by evidence O O O

Q12bvi. Other (e.g., sources of monetary support), specify (Q12bvii): O O O

Provide rows not specified in the list:
