# Supplementary material for: Harms in Systematic Reviews Paper 2: Methods used to assess harms are neglected in systematic reviews of gabapentin
Source: J Clin Epidemiol. Author manuscript; Available in PMC 2023 Mar 1. (PMC9875742; doi:10.1016/j.jclinepi.2021.10.024)
Supplement: 2 [file NIHMS1858687-supplement-2.docx]

**APPENDIX B – DATABASE SEARCH STRATEGIES**

Original search date: February 28, 2020

Search updated: September 17, 2020

**PubMed Search Strategy**

| **Search #** | **Text** | **Results** |
| --- | --- | --- |
| 1 | (Gabapentin[MESH] OR “Gamma-Aminobutyric Acid”[MESH:NoExp] OR Gabapentin[tiab] OR GABA[tiab] OR Gamma-Aminobutyric Acid[tiab] OR Neurontin[tiab] OR Cyclohexaneacetic Acid[tiab] OR Convalis[tiab] OR ApoGabapentin[tiab] OR NovoGabapentin[tiab] OR Neurotonin[tiab] OR Nupentin[tiab] OR Gralise[tiab] OR Kaptin[tiab] OR Keneil[tiab] OR Gabarone[tiab] OR Gabatin[tiab] OR Gabalept[tiab]) | 75492 |
| 2 | (Cochrane Database Syst Rev[Ta] OR Search[tiab] OR Systematic Review[Pt] OR Meta-Analysis[Pt] OR Medline[tiab] OR (Systematic[tiab] AND Review[tiab]) OR Meta-analysis[tiab] OR Meta-analyses[tiab]) | 526491 |
| 3 | 1 AND 2 | 1338 |
| 4 | Animals[MESH] NOT human[MESH] | 4673641 |
| 5 | 3 NOT 4 | 1185 |
| September 17, 2020 Search update | | + 64 |

**EMBASE Search Strategy**

| **Search #** | **Text** | **Results** |
| --- | --- | --- |
| 1 | ‘gabapentin’/exp OR ‘4 aminobutyric acid’/exp OR gabapentin:ti,ab OR GABA:ti,ab OR ‘gamma-aminobutyric acid’:ti,ab OR neurontin:ti,ab OR ‘cyclohexaneacetic acid’:ti,ab OR convalis:ti,ab OR apogabapentin:ti,ab OR novogabapentin:ti,ab OR neurotonin:ti,ab OR nupentin:ti,ab OR gralise:ti,ab OR kaptin:ti,ab OR keneil:ti,ab OR gabarone:ti,ab OR gabatin:ti,ab OR gabalept:ti,ab | 131732 |
| 2 | 'cochrane database syst rev':ta or (Search* or Medline or (Systematic and Review)):ab,ti,kw | 684530 |
| 3 | ([systematic review]/lim OR [meta analysis]/lim) | 326972 |
| 4 | 2 OR 3 | 784539 |
| 5 | 1 AND 4 | 4248 |
| 6 | (Animals/exp or invertebrate/exp or 'animal experiment'/exp or 'animal tissue'/exp or 'animal cell'/exp or nonhuman/exp) NOT (humans/exp) | 7289841 |
| 7 | 5 NOT 6 | 3834 |
| September 17, 2020 Search update | | + 145 |

**Epistemonikos Search Strategy**

title:("gabapentin" OR "neurontin" OR “convalis” OR “gralise” OR “neurotonin” OR “nupentin” OR “kaptin” OR “keneil” OR “gabarone” OR “gabatin” OR “gabalept” OR “gamma-aminobutyric acid”) OR abstract:( "gabapentin" OR "neurontin" OR “convalis” OR “gralise” OR “neurotonin” OR “nupentin” OR “kaptin” OR “keneil” OR “gabarone” OR “gabatin” OR “gabalept” OR “gamma-aminobutyric acid”)

Publication type: Systematic Review

Results: 477

September 17, 2020 Search Update: + 40 records

**Cochrane Database of Systematic Reviews Search Strategy**

“Gabapentin” in Cochrane Reviews

Results: 198

September 17, 2020 Search Update: + 7 records
