# Supplementary material for: Harms in Systematic Reviews Paper 2: Methods used to assess harms are neglected in systematic reviews of gabapentin
Source: J Clin Epidemiol. Author manuscript; Available in PMC 2023 Mar 1. (PMC9875742; doi:10.1016/j.jclinepi.2021.10.024)
Supplement: 1 [file NIHMS1858687-supplement-1.docx]

**APPENDIX A - Protocol**

**Methodologic overview of harms assessment among systematic reviews of gabapentin**

**ABSTRACT**

Current approaches to assessing harms of interventions produce an incomplete and biased understanding of the true balance of benefits to harms. Individual studies are usually underpowered with inadequate duration of follow up and too few events to detect all but the most common harms and no single study type can produce an accurate assessment of harms on its own. Systematic reviews and meta-analyses increase power to detect and quantify harms by synthesizing the results from independent studies. While recommendations do exist for how to conduct a systematic review of harms, the methods are consistently involving and variations in approaches taken by reviews for collecting and analyzing harms remain. We will evaluate the extent to which SRMAs of the same intervention are consistent in their approach and findings with regards to harms by conducting an overview of systematic reviews of gabapentin.

**BACKGROUND**

**Description of the problem and prior literature**

Current approaches to assessing harms of interventions produce an incomplete and biased understanding of the true balance of benefits to harms.^1–8^ Before an intervention can be approved for market, the Food and Drug Administration (FDA) requires that several studies, including clinical trials be conducted; the purpose of which is to determine an intervention’s overall safety and efficacy.^9^ Trials are excellent for determining causality, but have several challenges in the detection of harms including: highly selected populations and harms rarely being chosen as the primary outcomes.^10–14^ In contrast to RCTs, there are many different sources of observational data on harms (e.g., cohort studies, case-control studies, electronic medical records, insurance claims data, and spontaneous reporting systems) which can face issues of confounding and produce less strong evidence for causality but have some advantages for detecting harms over trials.^10–14^ Consequently, individual studies are usually underpowered with inadequate duration of follow up and too few events to detect all but the most common harms. No single study type can produce an accurate assessment of harms on its own.^11^

The challenge of assessing harms is further exacerbated by their methods of collection – most harms are collected non-systematically, through open-ended questions and spontaneous reporting, which increases their susceptibility to information bias as compared with efficacy outcomes and reduces comparability of results due to differences in ascertainment across trials and other study types ^6,7,15–18^ – and the systematic biases in their analysis and reporting.^15,19–21^

While the use of multiple data sources and types is desirable, how each type of evidence is complementary and dissimilar (or similar) to the others is still an active area of research. Systematic reviews and meta-analyses (the statistical combination of effects across studies) increase power to detect and quantify risks of harms by synthesizing the results from independent studies.^22–24^ However, meta-analysis is not a remedy for incomplete and biased ascertainment of harms at the trial level, nor a fix for under-reporting when it is based solely on published data: the validity of evidence syntheses are directly threatened by incomplete and improper reporting such as that caused by selection criteria.^4,24–27^ Selection criteria are the rules which define which adverse events are reported in a publication and can vary substantially between reports, even for reports of the same trial. Validity of meta-analyses can be increased by using individual participant data (IPD) from trials which circumvents issues caused by selection criteria, but IPD often remains unpublished and difficult to obtain for systematic reviewers.^28,29^

**Why it is important that we do this review**

There are multiple systematic reviews on gabapentin. To our knowledge, there are no systematic reviews that assess the consistency and methods with which harms are assessed and reported across systematic reviews of the same intervention. Multiple organizations have put forth recommendations for conducting systematic reviews of harms, but there are differences between these guidelines. Further, the variation in approaches taken by reviews for collecting and analyzing harms remains uncertain and understudied. Further, it is unclear how differences in approaches may affect the resulting summaries of harms.

**Description of the case study**

Gabapentin is an ideal case example to use for this methodologic overview because of its age as a regulated product. Gabapentin (Neurontin) was approved by the FDA in 1993 as adjunctive therapy for partial complex seizures. As gabapentin has been approved for 27 years, there are many primary studies that have been done to assess its benefits and risks, and subsequently there are also many systematic reviews. Additionally, gabapentin already has an established safety profile – supplemented from many different sources including randomized trials, observational studies, and surveillance systems – allowing us to focus on methods as opposed to a specific clinical question regarding its expected harms. A newer product would not likely have a sufficient number of reviews to allow generalized conclusions regarding systematic review methods. Furthermore, restricting our assessment to reviews for a specific drug will allow us to also assess how differing methods may affect the resulting syntheses and summaries of harms, as we expect reviews to reach similar conclusions regarding harms across conditions.

**Objectives**

To evaluate the extent to which SRMAs of the same intervention are consistent in their approach and findings with regards to harms by conducting an overview of systematic reviews of gabapentin for eleven conditions for which gabapentin is commonly prescribed.

**METHODS**

**Search methods for identification of reviews**

We will search PubMed, EMBASE, Epistemonikos, and the Cochrane Database of Systematic Reviews from 1990 to *Present* for SRs of gabapentin. Our original search strategy will be created for PubMed and subsequently translated to the other databases’ search syntax and use three of PICOTS components: indication (Population), drug (Intervention), and systematic review (Study type). We will apply a filter for study type (**Box 1**) developed from a search strategy that the Cochrane Eyes and Vision United States Satellite (CEV@US) uses to maintain a database of systematic reviews in the field of eyes and vision. We will not use any language restrictions in our search.

**Box 1. CEV@US PubMed filter for systematic reviews and meta-analyses**

(Cochrane Database Syst Rev[Ta] **OR** Search[tiab] **OR** Systematic Review[Pt] **OR** Meta-Analysis[Pt] **OR** Medline[tiab] **OR** (Systematic[tiab] **AND** Review[tiab]) **OR** Meta-analysis[tiab] **OR** Meta-analyses[tiab])

We will import all results into EndNote for de-duplication, and subsequently into Covidence for screening. We will perform screening in duplicate at two levels (title/abstract and full text) and resolve all disagreements through discussion. The complete search strategies can be found in Appendices A – D.

**Selection of reviews**

To be included in our overview review, studies must be systematic reviews or meta-analyses – either self-identified in the abstract or body of the review or by meeting the criteria for a systematic review as defined by the Institute of Medicine. We will exclude narrative reviews, scoping reviews, and other overview reviews because the methods for performing these studies are different from systematic reviews of primary studies.

**Box 2. Eligible conditions commonly prescribed gabapentin**

- postherpetic neuralgia
- epilepsy
- neuropathic pain
- seizure disorders
- restless leg syndrome
- vasomotor symptoms

(i.e., hot flashes)

- psychiatric disorders (bipolar disorder, attention deficit disorder, obsessive compulsive disorder, and post-traumatic stress disorder)
- migraine headaches
- post-operative pain
- fibromyalgia
- alcohol dependence

The review must be designed to specifically examine gabapentin as the intervention or comparator for any of eleven conditions commonly prescribed gabapentin (**Box 2**). Network meta-analyses or systematic reviews of multiple interventions will be included if they pre-specify gabapentin as an intervention of interest and meet all other inclusion criteria. We will accept reviews of gabapentin studied at any dose, duration of treatment, and form (i.e., generic vs. branded, and immediate-release vs. sustained-release). We will exclude reviews of other gabapentinoids (e.g., pregabalin, mirogabalin, gabapentin encarbil). We will also exclude any reviews which are designed to assess multiple treatment options and include gabapentin but which were not specifically designed to include gabapentin (e.g., non-specific reviews of all possible treatments for a specific condition or general reviews of an intervention vs. multiple comparators of which gabapentin is one) as the harms of gabapentin will be “confounded” by effects from other drugs or interventions.

The review must include an assessment of harms/adverse events/side effects/safety with results data from their included studies (note: this can be a general statement including that they found none (e.g., included studies did not report harms)). Reviews which do not include any studies on gabapentin will be excluded. We will document the number of reviews meet the eligibility criteria otherwise but which did not assess any harm or present any statements on harms data.

We will include only reliable reviews (assessed using a form developed by CEV@US – see ‘*Assessment of methodologic quality of reviews*’). The purpose of this is two-fold: to narrow our scope and to examine reviews that have a greater utility for practice. If a review itself is not reliable, then the presented evidence may not be complete (i.e., the search may not have captured all the relevant literature) or may be biased: the evidence may be inadequately assessed, inappropriately analyzed, or misrepresented.

In summary, our eligibility criteria are as follows:

- Systematic review of meta-analysis
- Designed to specifically examine gabapentin as the intervention or comparator (includes NMA or SRs which pre-specify gabapentin as one of multiple interventions of interest)
- Any of eleven conditions commonly prescribed gabapentin (Box 2)
- Must include an assessment of at least one harm with results data
- Review must be assessed as “reliable” using the CEV reliability tool (part of the data extraction form)

**Assessment of methodological quality of reviews**

To assess reliability, we adapted a data extraction form used by our team in previous studies.^30–35^ Data items on assessing the quality and methods for systematic reviews came from the Critical Appraisal Skills Programme (CASP),^36^ the Assessment of Multiple Systematic Reviews (AMSTAR),^37^ and the Preferred Reporting Items for Systematic reviews and Meta-Analyses (PRISMA).^38^

We classified systematic review as potentially “reliable” if the systematic review met the following methodologic criteria: (1) defined eligibility criteria for selection of individual studies, (2) conducted a comprehensive literature search for eligible studies, (3) assessed the risk of bias of the individual included studies using any method, (4) used appropriate methods for meta-analyses (criterion was only assessed if meta-analysis was performed), and (5) we observed concordance between the review findings and conclusions. We considered a systematic review “unreliable” when one or more of these criteria were not met. Definitions of the reliability assessment criteria are given in **Box 3**.

| **Box 3. Criteria for assessing the reliability of systematic reviews** | |
| --- | --- |
| **Criterion** | **Definition applied to systematic review reports** |
| Defined eligibility criteria | Described inclusion and/or exclusion criteria for eligible studies. |
| Conducted comprehensive literature search | Review authors (1) described an electronic search of two or more bibliographic databases; (2) used a search strategy comprising a mixture of controlled vocabulary and keywords; (3) reported using at least one other method of searching such as searching of conference abstracts; identified ongoing trials; complemented electronic searching by hand search methods (e.g., checking reference lists); and contacted included study authors or experts. |
| Assessed risk of bias of included studies | Used any method (e.g., scales, checklists, or domain-based evaluation) designed to assess methodologic rigor of included studies. |
| Used appropriate methods for meta-analysis | Used quantitative methods that (1) were appropriate for the study design analyzed (e.g., maintained the randomized nature of trials; used adjusted estimates from observational studies); (2) correctly computed the weight for included studies. |
| Observed concordance between review findings and conclusions | Authors’ reported conclusions were consistent with findings, provided a balanced consideration of benefits and harms, and did not favor a specific intervention if there was lack of evidence. |

**Data extraction and management**

From all reviews assessed as reliable and having at least one harm, we will extract data on the review itself with a focus on the general characteristics of the review and the methods used for assessing harms. We will use Systematic Review Data Repository to extract the data in duplicate from all included reviews. Specifically, the data we extract include:

Review characteristics:

- PICO (Population, Intervention, Comparators, Outcomes)
- Types and numbers of included studies
- Use of unpublished data and how it was obtained
- Were their harms pre-specified (confirmatory), exploratory, or a mix
  - Rationale for their decision (if provided)
- Report of following any specific guidance for harms
- Whether a protocol is available (if not online, request from authors)
  - If a protocol is available, does it address harms

Analysis methods *for harms*:

- Only qualitative/descriptive or quantitative (i.e., meta-analysis)
- If meta-analysis is performed:
  - The type of analysis (e.g., fixed/random effects, Odds Ratio/Risk Ratio/Risk Difference)
  - Which specific model used (e.g., inverse variance, Dersimian and Laird, Peto-OR, Mantel-Haenszel, Bayesian)
  - Specific methods for handling low counts (e.g., inclusion of zero-event studies, use of continuity correction
- Specific methods for handling missing data (e.g., ignore, last-value-carried-forward, some form of imputation)

Results and reporting *of harms*:

- Does the abstract include a statement about harms
  - If so, is the statement generic or does it mention specific harms
- Selection criteria used to report harms (i.e., do they report all harms they find in primary studies or some meeting their own applied criteria)
- The resulting profile of harms:
  - Specific harms that are presented in the review
  - Estimates of risk for each harm (if provided)
  - Time point of assessment for each harm (if provided)
- Discussion and interpretation of harms
  - Verbatim concluding statement about harms
  - Whether a general or specific statement is made regarding harms identified in the review
  - Whether any limitations regarding harms collection are discussed
  - Verbatim limitations of harms

**Analysis and synthesis**

We will not perform any meta-analyses in this systematic review as we expect the clinical heterogeneity between reviews with regards to population, interventions, and outcomes will be too great to permit the statistical combination of outcomes. We will qualitatively describe and compare the methods used across all studies which meet our inclusion criteria. We will create a table of study characteristics to present the above methods used in each study and we will summarize the approaches and methods used according to stage of production (e.g., how are reviews collecting harms; how are reviews synthesizing harms and, if performing meta-analyses, what assumptions and models are used; and how are reviews reporting harms). Additionally, we will assess the consistency of the synthesized harms by direct side-by-side comparison of reported harms and tabulating how many reviews included each harm that appears.

It may be that the profiles of harms differ between reviews due to the studies which they are including – selection criteria are inconsistent across reports for the same study and thus reviews may arrive at different answers depending on the sources for the studies that they include. We will assess if there is any overlap of primary studies between reviews and whether these reviews arrive at similar conclusions.

We may find disparate results for harms between reviews, including: half of the reviews not even considering examining harms; the half that do examine harms all using different definitions and pre-specifying different AEs to examine; or the measures of effect for common harms are all different, not only in magnitude but also direction.

The current guidelines for conducting systematic reviews of harms address different aspects, from searching to analysis and reporting (Table). It is unclear if these guidelines are followed in any capacity, although it is likely that these specific guidance documents will not be followed for many reviews as the earliest was released in 2008. If we find the differences in harms are too great between reviews, this will be a very serious finding as it may suggest that the use of non-standardized methods and reports included in any specific review influence the resulting harms to such as extent as to make them not generalizable beyond the included study samples. This finding would potentially serve as a stimulus to issue recommendations to journal editors to follow similar protocols for systematic reviews and meta-analyses as are currently followed for randomized clinical trials, namely: registration and protocol availability, and the following of established standards for searching, analysis, and reporting.

**Table**. Five systematic review guidance documents for harms

| **Guideline** | **Harms-specific guidance** |
| --- | --- |
| AHRQ – 2008 – *Methods Reference Guide for Effectiveness and CER: Assessing Harms When Comparing Medical Interventions* | - Assess all important harms, whenever possible. - Use multiple sources of information, including clinical experts and stakeholders, to identify important harms. Gather evidence on harms from a broad range of sources, including observational studies (including reporting systems), particularly when clinical trials are lacking; when generalizability is uncertain; or when investigating rare, long-term, or unexpected harms. - Assess Risk of Bias for Harms separately: Use consistent and precise terminology when reporting data on harms, and avoid terms implying causality unless causality is reasonably certain. Be cautious about drawing conclusions on harms when events are rare and estimates of risk are imprecise. Do not assume studies adequately assess harms because methods used to assess and report benefits are appropriate; rather, evaluate how well studies identify and analyze harms. - Include placebo-controlled trials, particularly for assessing uncommon or rare harms, but be cautious about relying on indirect comparisons to judge comparative risks and evaluate whether studies being considered for indirect comparisons meet assumptions for consistency of treatment effects. - Be careful conducting analyses of harms, but no structured guidance is presented. - Avoid inappropriate combining of data on harms, and thoroughly investigate inconsistent results |
| IOM – 2011 – *Finding What Works in Health Care: Standards for Systematic Reviews* | - Use observational studies in addition to clinical trials - Use unpublished data if it is possible to obtain - AE reporting systems may be a useful source of harms data |
| PRISMA-Harms – 2016 – *PRISMA harms checklist: improving harms reporting in systematic reviews* | - Contains four extension items that must be used in any systematic review addressing harms, irrespective of whether harms are analysed alone or in association with benefits: - Item 1—title: specifically mention "harms" or other related terms, or the harm of interest in the systematic review. - Item 14—synthesis of results: specify how zero events were handled, if relevant. - Item 18—study characteristics: define each harm addressed, how it was ascertained (eg, patient report, active search), and over what time period. - Item 21—synthesis of results: describe any assessment of possible causality. |
| FDA – 2018 – *Guidance for Industry: Meta-Analyses of Randomized Controlled Clinical Trials to Evaluate the Safety of Human Drugs or Biological Products* | - Focus is on trials and analysis; does not address observational studies or searching for studies which is the primary focus of the IOM and AHRQ guidance - High quality meta-analysis often requires unpublished data for harms; limiting meta-analysis to only published literature is hugely problematic - If the goal is confirmatory rather than exploratory, you need to use caution when including the trials that gave you the harms you are trying to confirm because of differences in ascertainment across trials. - No specific recommendations regarding handling multiplicity besides planning and pre-specification of meta-analyses - One approach to account for multiple testing and other sources of bias and error in meta-analyses of harms is to use a lower p-value - Do not use continuity corrections for zero-event trials or zero-event groups - Consider using difference measures instead of relative measures because differences are not as affected by zero-cell counts. Or else try Bayesian methods for meta-analysis - Fixed effects meta-analysis will have a better power, but random-effects is probably better for quantifying the risk itself - Conduct sensitivity analyses if possible (e.g., including/excluding specific trials, varying definitions for harms) |
| Cochrane – 2019 – *Chapter 19 (Adverse effects), Chapter 10 (Meta-analysis), Chapter 26 (Individual Participant Data)* | - Restriction to published data on adverse events is problematic - Multiple study types need to be considered - Many different sources, other than PubMed, MEDLINE, and EMBASE need to be searched - Addresses some challenges for assessing Risk of Bias for harms data - Look at unique adverse events: do not put AEs together into composites - Do not say events are “0” just because none were reported in the included studies - Regarding analysis of harms: - Difference methods are less susceptible to problems arising from 0 cell counts and they do not often require correction methods - If corrections must be used, non-fixed corrections (e.g., proportional to the reciprocal of the size of the contrasting study arm) are better than fixed - DerSimonian and Laird odds ratio and risk difference methods, and the Mantel-Haenszel odds ratio method using a 0.5 zero-cell correction (and really any inverse-variance method) are highly biased with rare events and should not be used for AE meta-analysis - Peto one-step OR is least biased and most powerful when event rates were below 1% - Mantel-Haenszel odds ratio without zero-cell corrections, logistic regression and an exact method are the best methods when event risk is above 1% - There are a number of different sources and initiatives that can be searched for IPD, and it is becoming more common to share - Two-stage meta-analysis is standard and much easier to achieve than one-stage IPD meta-analysis which requires mixed effects and multilevel regression modeling |

**Handling of heterogeneity**

As this review is an overview of methods, our focus is on the approaches used to synthesize harms among systematic reviews, rather than a specific clinical question. Consequently, we are casting a broad net with regards to our eligibility criteria to collect a more generalizable sample of reviews. We expect a large degree of clinical heterogeneity in our included reviews stemming from different doses of gabapentin (e.g., typical dosing ranges from 900mg to 3600mg per day), different durations of treatment (e.g., common study durations are between 4 to 12 weeks), different forms of gabapentin (e.g., generic vs. branded and immediate-release vs. sustained-release), and from the various conditions which we are accepting (**Box 2**).

We will not stratify our qualitative assessment of harms methodology by any of the afore mentioned sources of heterogeneity, as we see no reason why these methods should differ by any of these characteristics. Further, in accordance with the presentation of harms in the drug label for gabapentin, we do not expect the resulting profile of harms to differ by condition. Thus, we will not stratify our analyses of harms by condition. However, it is possible that the expected harms may differ depending on dose, duration, or form, and so we will assess the harms presented across all reviews and stratified by these three characteristics.

**List of Appendices**

APPENDIX A – Database Search Strategies

APPENDIX B – PRISMA Flow Diagram Shell

APPENDIX C – Table Shell: Study characteristics (by individual study, as in Cochrane reviews)

APPENDIX D – Table Shell: Methods for synthesizing harms

**REFERENCES**

1. Prasad V, Cifu A. Medical reversal: Why we must raise the bar before adopting new technologies. *Yale J Biol Med*. 2011;84:471-478.

2. Dwan K, Gamble C, Williamson PR, Kirkham JJ, Reporting Bias Group. Systematic review of the empirical evidence of study publication bias and outcome reporting bias - An updated review. *PLoS One*. 2013;8(7):e66844. doi:10.1371/journal.pone.0066844

3. Golder S, Loke YK, Wright K, Norman G. Reporting of adverse events in published and unpublished studies of health care interventions: A systematic review. *PLoS Med*. 2016;13(9):e1002127. doi:10.1371/journal.pmed.1002127

4. Kirkham JJ, Dwan KM, Altman DG, et al. The impact of outcome reporting bias in randomised controlled trials on a cohort of systematic reviews. *BMJ*. 2010;340:c365. doi:10.1136/bmj.c365

5. Wieseler B, Wolfram N, McGauran N, et al. Completeness of reporting of patient-relevant clinical trial outcomes: Comparison of unpublished clinical study reports with publicly available data. *PLoS Med*. 2013;10(10):e1001526. doi:10.1371/journal.pmed.1001526

6. Ioannidis JP, Lau J. Completeness of safety reporting in randomized trials: An evaluation of 7 medical areas. *JAMA*. 2001;285(4):437-443. doi:10.1001/jama.285.4.437

7. Ioannidis JP. Adverse events in randomized trials: Neglected, restricted, distorted, and silenced. *Arch Intern Med*. 2009;169(19):1737-1739. doi:10.1001/archinternmed.2009.313

8. Chan A-W, Hrobjartsson A, Haahr MT, Gøtzsche PC, Altman DG. Empirical evidence for selective reporting of outcomes in randomized trials. *JAMA*. 2004;291(20):2457-2465. doi:10.1001/jama.291.20.2457

9. Food and Drug Administration. The FDA’s drug review process: ensuring drugs are safe and effective. FDA Information for Consumers. https://www.fda.gov/Drugs/ResourcesForYou/Consumers/ucm143534.htm. Published 2018. Accessed December 5, 2018.

10. Hammad TA, Pinheiro SP, Neyarapally GA. Secondary use of randomized controlled trials to evaluate drug safety: A review of methodological considerations. *Clin Trials*. 2011;8(5):559-570. doi:10.1177/1740774511419165

11. Tsang R, Colley L, Lynd LD. Inadequate statistical power to detect clinically significant differences in adverse event rates in randomized controlled trials. *J Clin Epidemiol*. 2009;62:609-616. doi:10.1016/j.jclinepi.2008.08.005

12. Papanikolaou PN, Christidi GD, Ioannidis JP. Comparison of evidence on harms of medical interventions in randomized and nonrandomized studies. *J Can Med Assoc*. 2006;174(5):635-641. doi:10.1503/cmaj.050873

13. Chou R, Aronson N, Atkins D, et al. AHRQ series paper 4: assessing harms when comparing medical interventions: AHRQ and the effective health-care program. *J Clin Epidemiol*. 2010;63(5):502-512. doi:10.1016/j.jclinepi.2008.06.007

14. Vandenbroucke JP. When are observational studies as credible as randomised trials? *Lancet*. 2004;363:1728-1731. doi:10.1016/S0140-6736(04)16261-2

15. Ioannidis JP, Evans SJ, Gøtzsche PC, et al. Improving patient care better reporting of harms in randomized trials : An extension of the CONSORT statement. *Ann Intern Med*. 2014;141:781-788.

16. Mayo-Wilson E, Li T, Fusco N, et al. Cherry-picking by trialists and meta-analysts can drive conclusions about intervention efficacy. *J Clin Epidemiol*. 2017;91:95-110. doi:10.1016/j.jclinepi.2017.07.014

17. Mayo-Wilson E, Fusco N, Li T, Hong H, Canner JK, Dickersin K. Multiple outcomes and analyses in clinical trials create challenges for interpretation and research synthesis. *J Clin Epidemiol*. 2017;86:39-50. doi:10.1016/j.jclinepi.2017.05.007

18. Dickersin K, Mayo-Wilson E. Standards for design and measurement would make clinical research reproducible and usable. *Proc Natl Acad Sci*. 2018;115(11):2590-2594. doi:10.1073/pnas.1708273114

19. Altman DG, Moher D, Schulz KF. Harms of outcome switching in reports of randomised trials: CONSORT perspective. *BMJ*. 2017;356:j396. doi:10.1136/bmj.j396

20. Smyth R, Kirkham JJ, Jacoby A, Altman DG, Gamble C, Williamson P. Frequency and reasons for outcome reporting bias in clinical trials: Interviews with trialists. *BMJ*. 2010;341:c7153. doi:10.1136/bmj.c7153

21. Hodkinson A, Kirkham JJ, Tudur-Smith C, Gamble C. Reporting of harms data in RCTs: A systematic review of empirical assessments against the CONSORT harms extension. *BMJ Open*. 2013;3:e003436. doi:10.1136/bmjopen-2013-003436

22. Center for Drug Evaluation and Research. *Meta-Analyses of Randomized Controlled Clinical Trials to Evaluate the Safety of Human Drugs or Biological Products (DRAFT)*. Vol November. Washington, DC; 2018.

23. Hernandez A V., Walker E, Ioannidis JPA, Kattan MW. Challenges in meta-analysis of randomized clinical trials for rare harmful cardiovascular events: The case of rosiglitazone. *Am Heart J*. 2008;156(1):23-30. doi:10.1016/j.ahj.2008.03.002

24. Kirkham JJ, Riley RD, Williamson PR. A multivariate meta-analysis approach for reducing the impact of outcome reporting bias in systematic reviews. *Stat Med*. 2012;31:2179-2195. doi:10.1002/sim.5356

25. Zorzela L, Golder S, Liu Y, et al. Quality of reporting in systematic reviews of adverse events: Systematic review. *BMJ*. 2014;348:f7668. doi:10.1136/bmj.f7668

26. Saini P, Loke YK, Gamble C, Altman DG, Williamson PR, Kirkham JJ. Selective reporting bias of harm outcomes within studies: Findings from a cohort of systematic reviews. *BMJ*. 2014;349:g6501. doi:10.1136/bmj.g6501

27. Golder S, Loke YK, Wright K, Sterrantino C. Most systematic reviews of adverse effects did not include unpublished data. *J Clin Epidemiol*. 2016;77:125-133. doi:10.1016/j.jclinepi.2016.05.003

28. Tierney JF, Stewart LA, Clarke M. Chapter 26: Individual participant data. In: Higgins J, Thomas J, Chandler J, et al., eds. *Cochrane Handbook for Systematic Reviews of Interventions*. Version 6. Cochrane; 2019:643-658. doi:10.1002/9781119536604.ch26

29. Deeks JJ, Higgins JP, Altman DG. Chapter 10: Analysing data and undertaking meta‐analyses. In: Higgins J, Thomas J, Chandler J, et al., eds. *Cochrane Handbook for Systematic Reviews of Interventions*. Version 6. Cochrane; 2019:241-284. doi:10.1002/9781119536604.ch10

30. Golozar A, Chen Y, Lindsley K, et al. Identification and description of reliable evidence for 2016 American academy of ophthalmology preferred practice pattern guidelines for cataract in the adult eye. *JAMA Ophthalmol*. 2018;136(5):514-523. doi:10.1001/jamaophthalmol.2018.0786

31. Mayo-Wilson E, Ng SM, Chuck RS, Li T. The quality of systematic reviews about interventions for refractive error can be improved: A review of systematic reviews. *BMC Ophthalmol*. 2017;17:164. doi:10.1186/s12886-017-0561-9

32. Le JT, Qureshi R, Twose C, et al. Evaluation of systematic reviews of interventions for retina and vitreous conditions. *JAMA Ophthalmol*. 2019;137(12):1399-1406. doi:10.1001/jamaophthalmol.2019.4016

33. Yu T, Li T, Lee K, Friedman D, Dickersin K, Puhan M. Setting priorities for comparative effectiveness research on management on primary angle closure: A survey of Asia-Pacific clinicians. *J Glaucoma*. 2015;24(5):348-355. doi:10.1016/j.physbeh.2017.03.040

34. Lindsley K, Li T, Ssemanda E, Virgili G, Dickersin K. Interventions for age-related macular degeneration: Are practice guidelines based on systematic reviews? *Ophthalmology*. 2016;123(4):884-897. doi:10.1016/j.physbeh.2017.03.040

35. Li T, Vedula S, Scherer R, Dickersin K. What comparative effectiveness research is needed? A framework for using guidelines and systematic reviews to identify evidence gaps and research priorities. *Ann Intern Med*. 2012;156(5):367-377. doi:10.1038/jid.2014.371

36. Critical Appraisal Skills Programme (CASP). Critical Appraisal Skills Programme [Internet]. https://casp-uk.net/casp-tools-checklists. Published 2019. Accessed December 18, 2019.

37. Shea BJ, Grimshaw JM, Wells G a, et al. Development of AMSTAR: a measurement tool to assess the methodological quality of systematic reviews. *BMC Med Res Methodol*. 2007;7:10. doi:10.1186/1471-2288-7-10

38. Moher D, Liberati A, Tetzlaff J, Altman D, The PRISMA Group. Preferred reporting items for systematic reviews and meta-analyses: The PRISMA statement. *PLoS Med*. 2009;6(7):e1000097. doi:10.1371/journal.pmed.1000097

**APPENDIX A – DATABASE SEARCH STRATEGIES**

**PubMed Search Strategy [Search date: 28 Feb 2020]**

**REVISED (with Lori)**

| **Search #** | **Text** | **Results** |
| --- | --- | --- |
| 1 | (Gabapentin[MESH] OR “Gamma-Aminobutyric Acid”[MESH:NoExp] OR Gabapentin[tiab] OR GABA[tiab] OR Gamma-Aminobutyric Acid[tiab] OR Neurontin[tiab] OR Cyclohexaneacetic Acid[tiab] OR Convalis[tiab] OR ApoGabapentin[tiab] OR NovoGabapentin[tiab] OR Neurotonin[tiab] OR Nupentin[tiab] OR Gralise[tiab] OR Kaptin[tiab] OR Keneil[tiab] OR Gabarone[tiab] OR Gabatin[tiab] OR Gabalept[tiab]) | 75492 |
| 2 | (Cochrane Database Syst Rev[Ta] OR Search[tiab] OR Systematic Review[Pt] OR Meta-Analysis[Pt] OR Medline[tiab] OR (Systematic[tiab] AND Review[tiab]) OR Meta-analysis[tiab] OR Meta-analyses[tiab]) | 526491 |
| 3 | 1 AND 2 | 1338 |
| 4 | Animals[MESH] NOT human[MESH] | 4673641 |
| 5 | 3 NOT 4 | 1185 |
| September 17, 2020 Search update | | + 64 |

CAS Registry number: “60142-96-3”[rn]

**EMBASE Search Strategy [Search date: 28 Feb 2020]**

**REVISED (with Lori)**

| **Search #** | **Text** | **Results** |
| --- | --- | --- |
| 1 | ‘gabapentin’/exp OR ‘4 aminobutyric acid’/exp OR gabapentin:ti,ab OR GABA:ti,ab OR ‘gamma-aminobutyric acid’:ti,ab OR neurontin:ti,ab OR ‘cyclohexaneacetic acid’:ti,ab OR convalis:ti,ab OR apogabapentin:ti,ab OR novogabapentin:ti,ab OR neurotonin:ti,ab OR nupentin:ti,ab OR gralise:ti,ab OR kaptin:ti,ab OR keneil:ti,ab OR gabarone:ti,ab OR gabatin:ti,ab OR gabalept:ti,ab | 131732 |
| 2 | 'cochrane database syst rev':ta or (Search* or Medline or (Systematic and Review)):ab,ti,kw | 684530 |
| 3 | ([systematic review]/lim OR [meta analysis]/lim) | 326972 |
| 4 | 2 OR 3 | 784539 |
| 5 | 1 AND 4 | 4248 |
| 6 | (Animals/exp or invertebrate/exp or 'animal experiment'/exp or 'animal tissue'/exp or 'animal cell'/exp or nonhuman/exp) NOT (humans/exp) | 7289841 |
| 7 | 5 NOT 6 | 3834 |
| September 17, 2020 Search update | | + 145 |

**Epistemonikos Search Strategy [Search date: 28 Feb 2020]**

title:("gabapentin" OR "neurontin" OR “convalis” OR “gralise” OR “neurotonin” OR “nupentin” OR “kaptin” OR “keneil” OR “gabarone” OR “gabatin” OR “gabalept” OR “gamma-aminobutyric acid”) OR abstract:( "gabapentin" OR "neurontin" OR “convalis” OR “gralise” OR “neurotonin” OR “nupentin” OR “kaptin” OR “keneil” OR “gabarone” OR “gabatin” OR “gabalept” OR “gamma-aminobutyric acid”)

Publication type: Systematic Review

Results: 477

September 17, 2020 Search Update: + 40

**Cochrane Database of Systematic Reviews Search Strategy [Search date: 28 Feb 2020]**

“Gabapentin” in Cochrane Reviews

Results: 198

September 17, 2020 Search Update: + 7

**APPENDIX B – Shell PRISMA Flow Diagram**

PubMed

(n = 1185)

EMBASE

(n = 3834)

Epistemonikos

(n = 477)

Cochrane

(n = 198)

Results from all databases

(n = 5694)

**Initial search**

**Duplicates removed**

Auto [EndNote/Covidence] (n = 1443)

Hand [Covidence] (n = 94)

**Inclusion**

Reliable reviews of gabapentin

(n = 67)

**Screening**

Unreliable (n = 86)

Add-on therapy (n = 3)

Records after full-text screening

(n = 163)

Reviews assessed for reliability

(n = 156)

Unique records screened at title/abstract

(n = 4157)

Full-text records assessed

(n = 487)

**Reasons for exclusion**

Not relevant (n = 3480)

Wrong condition (n = 92)

Pregabalin (n = 74)

Gabapentin as adjunctive (n = 24)

**Reasons for exclusion**

Not a SRMA (n = 90)

Not designed for gabapentin (n = 80)

Abstract only (n = 77)

No results for harms (n = 26)

No studies on gabapentin (n = 11)

Other (duplicate, withdrawn, wrong condition, adjunctive therapy, protocol for ongoing review) (n = 40)

**Original search February 28, 2020; updated September 17, 2020 Reasons for exclusion adjusted for clarity; updated February 9, 2020**

Reliable reviews of gabapentin

(n = 70)

Unreliable (n = 87)

Add-on therapy (n = 4)

Identified from included review (n = 1)

Records after full-text screening

(n = 169)

Reviews assessed for reliability

(n = 161)

**Inclusion**

**Screening**

Results from all databases

(n = 5950)

**Reasons for exclusion**

Not a SRMA (n = 92)

Not designed for gabapentin (n = 85)

Abstract only (n = 77)

No results for harms (n = 26)

No studies on gabapentin (n = 11)

Other (duplicate, withdrawn, wrong condition, adjunctive therapy, protocol for ongoing review) (n = 41)

**Reasons for exclusion**

Not relevant (n = 3624)

Wrong condition (n = 95)

Pregabalin (n = 75)

Gabapentin as adjunctive (n = 26)

Full-text records assessed

(n = 500)

Unique records screened at title/abstract

(n = 4320)

**Duplicates removed**

Auto [EndNote/Covidence] (n = 1536)

Hand [Covidence] (n = 94)

**Initial search**

Cochrane

(n = 205)

Epistemonikos

(n = 517)

EMBASE

(n = 3979)

PubMed

(n = 1249)

Reliable reviews of gabapentin

(n = 70)

Unreliable (n = 87)

**Inclusion**

EMBASE

(n = 3979)

Epistemonikos

(n = 517)

Cochrane

(n = 205)

Identified from full-text review (n = 1)

**Reasons for exclusion**

Not a SRMA (n = 92)

Not designed for gabapentin (n = 85)

Abstract only (n = 74)

No results for harms (n = 26)

No studies on gabapentin (n = 11)

Other (duplicate, withdrawn, wrong condition, adjunctive therapy, protocol for ongoing review, journal article unobtainable) (n = 48)

**Screening**

Records after full-text screening

(n = 165)

Reviews assessed for reliability

(n = 157)

Results from all databases

(n = 5950)

**Reasons for exclusion**

Not relevant (n = 3624)

Wrong condition (n = 95)

Pregabalin (n = 75)

Gabapentin as adjunctive (n = 26)

Full-text records assessed

(n = 500)

Unique records screened at title/abstract

(n = 4320)

**Duplicates removed**

Auto [EndNote/Covidence] (n = 1536)

Hand [Covidence] (n = 94)

**Initial search**

PubMed

(n = 1249)

**APPENDIX C - Table shell. Study characteristics (presented for each included review)**

| **Review ID - [Last name Date]** | | |
| --- | --- | --- |
| **Review Characteristic** |  | |
| Population (i.e., condition) | “Verbatim description of population” | |
| Intervention | Dose, formulation, etc. of intervention | |
| Comparator | Dose, formulation, etc. of comparison | |
| Outcome (Primary) | “Verbatim primary outcome” | |
| Types of included studies (* indicates study type included for gabapentin) | RCT / CCT / Cohort / Case-control / Surveillance system / Other | |
| Number of gabapentin studies (participants) \| Total number of included studies (participants) | # gabapentin studies (# GBP participants) \| # total studies (# total participants) | |
| Approach to defining harms | Confirmatory / Exploratory / Hybrid | |
| Approach to assessing harms | Qualitative / Quantitative / Both | |
| Specific guidance followed for harms | Cochrane / FDA / PRISMA-Harms / IOM/ AHRQ / Other | |
| Proportion of words devoted to harms | (# words in body of review describing harms) / (total words in body of review) | |
| Selection criteria used to report harms | Yes / No (presented all they identified) / Unclear | |
| Summary statement on harms | “Verbatim statement on harms” | |
| Profile of harms identified and synthesized in review  *Estimates provided if reported, otherwise ‘NR’ ^A^* | Absolute # occurrences  (n) | Estimate of risk  (%) |
| Harm 1 |  |  |
| Harm 2 |  |  |
| Harm 3 |  |  |
| … |  |  |
| Harm n |  |  |

A – If a review reported an estimate for the number of occurrences of a harm across studies and/or the estimate of risk, that value was extracted. If a harm was mentioned in the review but no quantitative estimate was reported, that harm is noted in the review’s profile with “NR” (Not reported) for the estimates. If no specific harms are mentioned in the review (i.e., the summary statement indicates that none were found or the harms assessment was general), the

**APPENDIX D - Table shell. Methods for assessing harms among __ systematic reviews of gabapentin**

| **Harms assessment** | **n** | **(%)** |
| --- | --- | --- |
| **Review included unpublished data** |  |  |
| Yes | _ | (_) |
| No | _ | (_) |
| Not reported | _ | (_) |
| **Approach to assessing harms** |  |  |
| Confirmatory | _ | (_) |
| Exploratory | _ | (_) |
| Hybrid approach | _ | (_) |
| **Followed specific guidance for harms** |  |  |
| Yes | _ | (_) |
| Cochrane | _ |  |
| FDA | _ |  |
| PRISMA – Harms | _ |  |
| AHRQ | _ |  |
| IOM | _ |  |
| Other | _ |  |
| No | _ | (_) |
| Not reported | _ | (_) |
| **Analysis methods for harms** ^a^ |  |  |
| Type of “harm” analyzed |  |  |
| Separate and specific harms | _ | (_) |
| Grouped specific harms | _ | (_) |
| Any non-specific harms | _ | (_) |
| Drop-out due to harms | _ | (_) |
| Other | _ | (_) |
| Qualitative (i.e., descriptive) | _ | (_) |
| Quantitative (i.e., meta-analysis) | _ | (_) |
| Measure of effect |  |  |
| Odds Ratio | _ | (_) |
| Risk Ratio | _ | (_) |
| Risk Difference | _ | (_) |
| Number Needed to Harm | _ | (_) |
| Other | _ | (_) |
| Type of analysis |  |  |
| Fixed effects | _ | (_) |
| Random effects | _ | (_) |
| Not reported | _ | (_) |
| Analysis model |  |  |
| Inverse variance | _ | (_) |
| Dersimonian and Laird | _ | (_) |
| Peto-OR | _ | (_) |
| Mantel-Haenszel | _ | (_) |
| Bayesian | _ | (_) |
| Other | _ | (_) |
| Not reported | _ | (_) |
| Inclusion of zero-event studies |  |  |
| Yes | _ | (_) |
| No | _ | (_) |
| Not reported | _ | (_) |
| Use of continuity correction |  |  |
| Yes | _ | (_) |
| No | _ | (_) |
| Not reported | _ | (_) |
| Handling of missing data |  |  |
| Ignore (i.e., complete case) | _ | (_) |
| Last value carried forward | _ | (_) |
| Simple imputation | _ | (_) |
| Multiple imputation | _ | (_) |
| Not reported | _ | (_) |
| **Reporting of harms** |  |  |
| Abstract includes statement on harms |  |  |
| Yes | _ | (_) |
| Specific statement | _ |  |
| General statement | _ |  |
| No | _ | (_) |
| Use of selection criteria |  |  |
| No – reported all harms identified | _ | (_) |
| Yes – reported a subset of harms identified | _ | (_) |
| Unclear – no statement on use of criteria | _ | (_) |

a No analysis element is mutually exclusive as reviews may take different approaches for multiple harms, from assessing some qualitatively and others quantitatively, to using different quantitative models for different harms.

**LIST OF PROTOCOL REVISIONS/MODIFICATIONS**

**March 12, 2020 –** Added “Table. Five systematic review guidance documents for harms” for explicit comparison of the existing recommendations regarding methods for conducting systematic reviews of harms. More comprehensive and clear than the earlier summary statement about there being some similarities across resources.

**March 20, 2020 –** Updated and clarified criteria regarding harms results in reviews. Originally: “*The review must include an assessment of at least one harm with results data (i.e., a review that reports a plan to study harms but included studies do not present any data on harms would be excluded).*” Updated and clarified to: “*Must include an assessment of harms/adverse events/side effects/safety with results data from their included studies (note: this can be a general statement including that they found none (e.g., included studies did not report harms)).*” This change and clarification was made at the start of title/abstract screening as screeners considered that studies conducting a complete assessment of harms may make a general summary statement about harms that is non-specific and which would technically be excluded by the original criteria, even if an assessment of harms was actually conducted (e.g., assessing the risk of “any non-specific event” or assessing the risk of loss-to-follow-up due to harm). It was decided that all studies with a statement about harms assessment should be included. We have kept the original part of the wording regarding keeping track of reviews which do not include a statement about harms: “*We will document the number of reviews meet the eligibility criteria otherwise but did not assess any harm or present any statements on harms data*.”

**April 8, 2020 –** Updated search numbers in PRISMA flow diagram

**May 20, 2020 –** Updated screening numbers in PRISMA flow diagram

**June 17, 2020 –** Added additional elements for extraction to table shells based on things noticed during full-text screening (i.e., needing a verbatim “summary statement of harms” for the review and adding the “type of harm” analyzed for the review)

**August 19, 2020 –** Added the number of reliable reviews and removed “proportion of manuscript devoted to harms” as something assessed. After a trial, this was too difficult to do accurately and would not add much quantitatively to our assessment; qualitatively we can see that reviews dedicate only a small proportion of their results and discussion to harms unless they are specifically designed for harms.
